# Prevalence of *Campylobacter* and *Salmonella* in African food animals and meat: A systematic review and meta-analysis

**DOI:** 10.1016/j.ijfoodmicro.2019.108382

**Published:** 2020-02-16

**Authors:** Kate M. Thomas, William A. de Glanville, Gary C. Barker, Jackie Benschop, Joram J. Buza, Sarah Cleaveland, Margaret A. Davis, Nigel P. French, Blandina T. Mmbaga, Gerard Prinsen, Emmanuel S. Swai, Ruth N. Zadoks, John A. Crump

**Affiliations:** a Centre for International Health, Dunedin School of Medicine, University of Otago, Dunedin, New Zealand; b Kilimanjaro Clinical Research Institute, Good Samaritan Foundation, Moshi, United Republic of Tanzania; c Institute of Biodiversity Animal Health and Comparative Medicine, College of Medical Veterinary & Life Sciences, University of Glasgow, Glasgow, United Kingdom; d Norwich, United Kingdom; e mEpiLab, Massey University, Palmerston North, New Zealand; f School of Life Sciences and Bio-Engineering, Nelson Mandela African Institution of Science and Technology, Arusha, United Republic of Tanzania; g Paul G. Allen School for Global Animal Health, Washington State University, Pullman, WA, United States of America; h New Zealand Food Safety Science and Research Centre, New Zealand; i School of People, Environment and Planning, Massey University, Palmerston North, New Zealand; j State Department of Veterinary Services, Ministry of Livestock and Fisheries, Dodoma, United Republic of Tanzania

**Keywords:** Africa, *Campylobacter*, Food animals, Food safety, Prevalence, *Salmonella*

## Abstract

**Background:**

*Campylobacter* and *Salmonella*, particularly non-typhoidal *Salmonella*, are important bacterial enteric pathogens of humans which are often carried asymptomatically in animal reservoirs. Bacterial foodborne infections, including those derived from meat, are associated with illness and death globally but the burden is disproportionately high in Africa. Commercial meat production is increasing and intensifying in many African countries, creating opportunities and threats for food safety.

**Methods:**

Following Preferred Reporting Items for Systematic Reviews and Meta-analyses (PRISMA) guidelines, we searched six databases for English language studies published through June 2016, that reported *Campylobacter* or *Salmonella* carriage or infection prevalence in food animals and contamination prevalence in food animal products from African countries. A random effects meta-analysis and multivariable logistic regression were used to estimate the species-specific prevalence of *Salmonella* and *Campylobacter* and assess relationships between sample type and region and the detection or isolation of either pathogen.

**Results:**

Seventy-three studies reporting *Campylobacter* and 187 studies reporting *Salmonella* across 27 African countries were represented. Adjusted prevalence calculations estimate *Campylobacter* detection in 37.7% (95% CI 31.6–44.3) of 11,828 poultry samples; 24.6% (95% CI 18.0–32.7) of 1975 pig samples; 17.8% (95% CI 12.6–24.5) of 2907 goat samples; 12.6% (95% CI 8.4–18.5) of 2382 sheep samples; and 12.3% (95% CI 9.5–15.8) of 6545 cattle samples. *Salmonella* were detected in 13.9% (95% CI 11.7–16.4) of 25,430 poultry samples; 13.1% (95% CI 9.3–18.3) of 5467 pig samples; 9.3% (95% CI 7.2–12.1) of 2988 camel samples; 5.3% (95% CI 4.0–6.8) of 72,292 cattle samples; 4.8% (95% CI 3.6–6.3) of 11,335 sheep samples; and 3.4% (95% CI 2.2–5.2) of 4904 goat samples. ‘External’ samples (e.g. hide, feathers) were significantly more likely to be contaminated by both pathogens than ‘gut’ (e.g. faeces, cloaca) while meat and organs were significantly less likely to be contaminated than gut samples.

**Conclusions:**

This study demonstrated widespread prevalence of *Campylobacter* species and *Salmonella* serovars in African food animals and meat, particularly in samples of poultry and pig origin. Source attribution studies could help ascertain which food animals are contributing to human campylobacteriosis and salmonellosis and direct potential food safety interventions.

## Highlights

• *Campylobacter* prevalence data was compiled from 14 African countries.
• *Salmonella* prevalence data was compiled from 27 African countries.
• *Campylobacter* and *Salmonella* were most prevalent in poultry and pig samples.
• *C. jejuni* was the most predominant *Campylobacter* species except in pigs where *C. coli* predominates.
• *S. enterica* serovar Typhimurium was the most commonly identified *Salmonella* serovar.

## 1 Introduction

*Campylobacter* and non-typhoidal *Salmonella* (NTS) are bacterial enteric pathogens associated with food animal reservoirs. They are transmitted to humans predominantly by contaminated food and water. Foodborne zoonoses, including those caused by *Campylobacter* and NTS, are recognised by the World Health Organization (WHO) as important causes of human illness and death worldwide (Havelaar et al., 2015). It is estimated that *Campylobacter* are responsible for >95 million foodborne illnesses and >21,000 deaths and NTS for >78 million foodborne illnesses and >59,000 deaths globally (Havelaar et al., 2015). The burden of bacterial foodborne disease, including disease caused by *Campylobacter* and NTS, is disproportionately higher in African regions compared with other parts of the world, with the number of Disability Adjusted Life Years (DALYs) per 100,000 exceeding that of other global regions (Havelaar et al., 2015).

While *Campylobacter* and NTS infections are usually self-limiting in healthy humans, complicated disease may develop in some. Bacteraemia and immunological syndromes such as reactive arthritis have been linked to both pathogens (Carter and Hudson, 2009; Sandhu and Paul, 2014) while Guillain-Barré syndrome is associated with *Campylobacter* infection (Esan et al., 2017, World Health Organization (WHO), 2012). For both *Campylobacter* and NTS, bacteraemia is more common among children, the elderly, and immunocompromised persons, especially those with HIV/AIDS (Food and Drug Administration (FDA), 2012). Almost 70% of the global HIV burden is in sub-Saharan Africa (Joint United Nations (UN) Programme on HIV/AIDS, 2017). An increased risk of NTS bacteraemia has also been linked with recent or current malaria, and malnutrition (Feasey et al., 2012).

*Campylobacter* are usually a non-pathogenic component of the gastrointestinal tract microbiota of livestock such as cattle, pigs, and sheep, with poultry considered to be the major reservoir, particularly of *C. jejuni* (Sahin et al., 2002; Skarp et al., 2016). Similarly, NTS have been isolated from the gastrointestinal tract of birds and mammals, including poultry and livestock (Barrow et al., 1988; Ellis, 1969; Gay et al., 1994). The transmission of potentially pathogenic *Campylobacter* or NTS from animal hosts to humans is predominantly via faecally contaminated food and water. However, humans may be infected by contact with live animals and environments contaminated with animal faeces and subsequent incidental ingestion of pathogens (Cummings et al., 2012, World Health Organisation (WHO), 2017). During processing for meat, animal gut microbiota and associated pathogens from the caecum, cloaca, intestines, or faeces may be transferred directly to the carcass or meat and organ surfaces. Transfer may also be indirect, such as from human hands and equipment. Both pathways may result in transfer of gastro-intestinal flora to meat and organ surfaces or to external animal surfaces such as feathers, fleece, hide, and skin. Such transfer is associated with increased risk for human infection from consumption of contaminated meat and cross-contamination of other foods. Uncooked produce may also be a direct source of human infection with zoonotic pathogens through contamination by animal faeces or untreated irrigation water (Food and Drug Administration (FDA), 2012).

Meat production is central to livelihoods in many African countries, with meat from livestock and poultry being a key protein source in subsistence communities (OECD/FAO, 2016). In many low-resource settings, industrialisation, urbanisation, and the shift from planned to market economies are leading to rapid changes in the way that food is produced, distributed, sold, and consumed (Carron et al., 2018; Grace, 2017). Such market-driven changes within agricultural production towards wider distribution networks, centralised processing, larger-scale and more intensive systems, have been linked to the emergence of zoonotic diseases (Jones et al., 2013) and the potential impact on food safety within low- and middle-income countries is increasingly recognised (World Health Organisation (WHO) Regional Office for Africa, 2017). Data on key bacterial pathogens in the meat production pathway in Africa are limited and are not currently available in aggregate form. To inform food safety policy and to identify data gaps, we undertook a systematic review on *Campylobacter* and *Salmonella* prevalence in food animals and meat in Africa.

## 2 Methods

### 2.1 Study design and systematic review protocol

References were sought and identified following the Preferred Reporting Items for Systematic Reviews and Meta-analyses (PRISMA) guidelines (Moher et al., 2009) (Supplementary File 1 checklist). Studies were searched in African Index Medicus (AIM), CABI Global Health, Proquest Health and Medicine, PubMed, Scopus, and Web of Science. Search terms are listed in Table 1 and no date restrictions were applied. The last search took place on 21 July 2016.

### 2.2 Search strategy

Article titles and abstracts were reviewed for suitability for inclusion by KMT. They were selected for full text review if the studies investigated *Campylobacter* or *Salmonella*, reported on samples collected from food animals, their organs or meat, and data collection took place in African regions or countries as defined by the United Nations (UN) statistics division (United Nations (UN) Statistics Division, 2016). Full text articles were reviewed independently by two authors (KMT, WdG) to determine if each article met pre-determined inclusion and exclusion criteria (Supplementary File 2). Articles were included for full text review if the full text article could be retrieved, if it reported primary data, if the article reported isolation by culture or detection by PCR of *Campylobacter* or *Salmonella*, in individual food animals or meat products, regardless of laboratory methods used, if the prevalence of contamination with either pathogen could be calculated from information available in the paper, and if the materials tested represented ‘gut’, ‘external’, or meat and organ samples from individual animals. Gut samples were defined as caecal, intestinal, cloacal contents and faeces, which have the potential to contaminate ‘external’ and meat and organ samples from individual animals. External samples were defined as those from the exterior of individual animals, including hide, skin, or feathers. Meat and organ samples included bile, lymph nodes, raw organs, or meat. Serological studies were excluded. Studies were excluded if the numerator (i.e. number positive) and denominator (i.e. number tested) information were not reported at the species and sample type level. Studies were excluded if samples were frozen at the time of sample collection to avoid underestimates of prevalence data, particularly of *Campylobacter*, as a result of loss of viable cells due to freezing. Studies were excluded if they were solely from sick animals, from free-living wildlife, or from farmed game animals. Studies were also excluded if they were in a language other than English. When required, a third author (JAC) served as tiebreaker, independently reviewing articles to resolve disagreement between the two primary reviewers.

### 2.3 Data extraction

From each included article, we extracted information on source food animal species, sample type, the total number of samples tested. The number of *Campylobacter* or *Salmonella* isolated or detected was extracted to determine pathogen prevalence. Sample location data, including UN statistics division African geographic region (United Nations (UN) Statistics Division, 2016), country, administrative level, city, town or village, and Global Positioning System (GPS) coordinates were extracted from articles when reported. Where available, data were recorded on the prevalence of individual *Campylobacter* species, and *Salmonella enterica* serovars or serogroups, as per standards of the WHO Collaborating Centre for Reference and Research on *Salmonella* (Grimont and Weill, 2007). During data extraction, elements of sample selection, sample handling, and laboratory methods were noted, including length of study, conditions and time of transport to laboratory, amount of sample tested, media used, and temperature and gas conditions of incubation. A formal bias assessment was established (Supplementary Table 1), assigning low (L), moderate (M), high (H), unknown (U), implied (I), yes (Y), no (N), or not applicable (NA) to each potential introduction of bias. The bias elements considered in the formal assessment relating to sample selection and handling were study length, temperature and time of transport to the laboratory. The bias elements relating to laboratory testing were amount of sample tested, type of isolation or detection methods, incubation conditions, and the quality of the serotyping methods used. An overall assessment of low, moderate or high risk of bias was assigned to each included article.

### 2.4 Data analysis

Prevalence estimates were calculated from pooled data for each pathogen by livestock species, geographic region and sample type, and for each geographic region and sample type by livestock species. An inverse variance approach with study ID as a random effect was used to derive weighted prevalence estimates (Marín-Martínez and Sánchez-Meca, 2010). In the presence of small numbers of total samples for some studies, the logit transformation was used (Barendregt et al., 2013). Between study heterogeneity was quantified using the I^2^ statistic, which provides an estimate of the percentage of total variation between studies that is due to prevalence differences rather than to chance variation (Higgins et al., 2003). Eighty percent prediction intervals (80% PI) were derived to provide an estimate of the interval between which the prevalence of a future study of *Campylobacter* or *Salmonella* prevalence could be expected to fall with 80% probability (IntHout et al., 2016). Weighted average prevalence estimates, their 95% confidence intervals (95% CI), 80% PI and the I^2^ statistic were derived using the *meta* package (Schwarzer, 2007) in the R statistical environment, version 3.4.2. (http://cran.r-project.org/).

**Table 1:** Full search strategies for database searches, date searched, database name, and number of articles retrieved systematic review of prevalence of *Campylobacter* and *Salmonella* in African food animals and meat, 1953–2016.

Date Search performed	Database	Number of articles retrieved	Search string/terms and limits
11 Jul 16	Africa Index Medicus	46	Campylobacter OR Salmonella
21 Jul 16	CABI Global Health	2251	All = (Search #1) AND All = (Search #2) AND All = (Search #3)
11 Jul 16	ProQuest	774	Anywhere = (Search #1) AND All = (Search #2) AND All = (Search #3)
11 Jul 16	PubMed	1423	All = (Search #1) AND All = (Search #2) AND All = (Search #3)
11 Jul 16	Scopus	2059	Abstract, Title, Keyword = (Search #1) AND Abstract, Title, Keyword = (Search #2) AND Abstract, Title, Keyword = (Search #3) in the categories of Life Sciences and Health Sciences
11 Jul 16	Web of Science	1051	TOPIC = (Search #1) AND TOPIC = (Search #2) AND TOPIC = (Search #3)
		Where:	
		Search #1	(Campylobacter*) OR (Salmonell*)
		Search #2	(cattle) OR (cow) OR (bull) OR (beef) OR (heifer) OR (steer) OR (bovine) OR (calf) OR (calves) OR (sheep) OR (mutton) OR (hogget) OR (lamb) OR (ovine) OR (goat) OR (caprine) OR (chicken) OR (avian) OR (poultry) OR (hen) OR (chick) OR (broiler) OR (layer) OR (pork) OR (porcine) OR (pig) OR (camel) OR (offal) OR (food) OR (meat)
		Search #3	(Africa*) OR (algeria) OR (angola) OR (benin) OR (botswana) OR (burkina faso) OR (burundi) OR (cameroon) OR (cape verde) OR (central african republic) OR (chad) OR (comoros) OR (congo) OR (cote d'ivoire) OR (ivory coast) OR (democratic republic of the congo) OR (zaire) OR (djibouti) OR (egypt) OR (equatorial guinea) OR (eritrea) OR (ethiopia) OR (gabon) OR (gambia) OR (ghana) OR (guinea) OR (guinea-bissau) OR (kenya) OR (lesotho) OR (liberia) OR (libya) OR (madagascar) OR (malawi) OR (mali) OR (mauritania) OR (mauritius) OR (mayotte) OR (morocco) OR (mozambique) OR (namibia) OR (niger) OR (nigeria) OR (reunion) OR (rwanda) OR (saint helena) OR (sao tome and principe) OR (senegal) OR (seychelles) OR (sierra leone) OR (somalia) OR (south africa) OR (south sudan) OR (sudan) OR (swaziland) OR (tanzania) OR (togo) OR (tunisia) OR (uganda) OR (western sahara) OR (zambia) OR (zimbabwe)

Mixed effects logistic regression was used to explore predictors of *Campylobacter* and *Salmonella* infection or colonisation and contamination. Publication ID was included as a random effect and geographic region, sample type (i.e. ‘gut’, ‘external’, ‘meat and organ’) and species type were included as fixed effects. Data were then disaggregated by host species and separate mixed effects logistic regression models constructed for each pathogen for camels, cattle, goats, pigs, poultry, and sheep. Host species-specific models included publication ID as a random effect and sample type and geographic region as fixed effects. Where data were available, Northern Africa was used as the referent region and ‘gut’ as the baseline sample type. Deviations are stated when chosen referent baselines did not have data. Poultry was used as the baseline for species type and compared to pigs and a combined category containing all other species (buffalo, camels, cattle, goats, and sheep). The overall contribution of each fixed effect to model fit was assessed using a likelihood ratio test. In addition to the I^2^ statistic described above, between study heterogeneity in prevalence was quantified for the overall logistic regression model and species-specific logistic regression models using the median odds ratio (MOR). This statistic represents the median value of the odds ratio when comparing group (study) level residuals from randomly selected pairs of samples from different studies (Larsen and Merlo, 2005). It can be considered to provide an indication of the magnitude of the difference in odds of animal infection or carriage or sample contamination when comparing two studies: where there is little between study variation, the MOR would be close to one. Mixed effects logistic regression models were constructed using the *lme4* package (Bates et al., 2015) in R.

## 3 Results

**Fig. 1:**
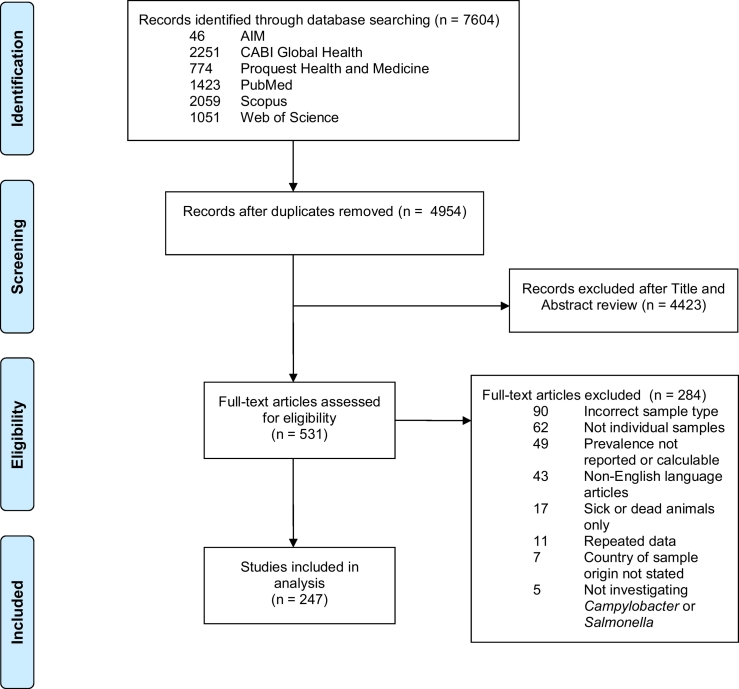
PRISMA flowchart showing identification, screening, and selection of eligible articles for inclusion in systematic review, 1953–2016.

**Fig. 2:**
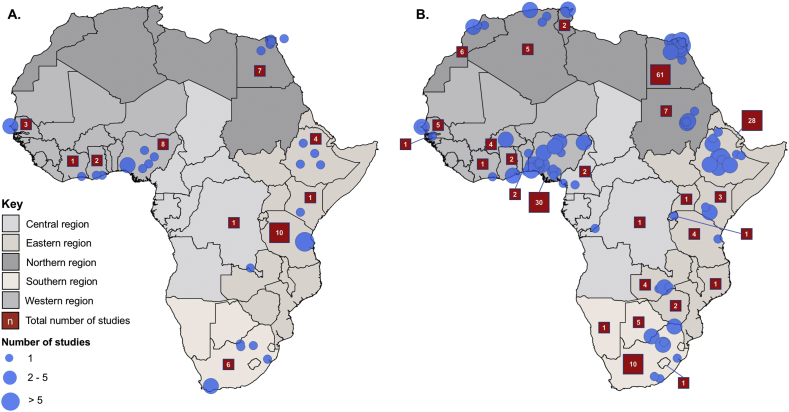
Map showing location of included studies describing (A) *Campylobacter* and (B) *Salmonella* contamination prevalence where geographic information was available (blue circles), 1953–2016. (For interpretation of the references to colour in this figure legend, the reader is referred to the web version of this article.)

After removing duplicate articles from the searches of six selected databases, 4954 articles were available for title and abstract screening. Of these 531 (10.7%) were identified as potentially relevant and 247 (5.0%) were eligible for inclusion after full text review (Fig. 1). Sixty articles from 14 countries reported prevalence data on *Campylobacter*, 174 articles from 27 countries reported prevalence data on *Salmonella,* and 13 articles from eight countries reported prevalence data on both pathogens. No prevalence data were excluded as a result of the quality assessment. Table 2 shows the decades in which the sampling began in each study. The geographic location of included studies is represented in Fig. 2. The number of studies from each country and the animal species investigated are listed in Table 3.

**Table 2:** Numbers of articles on *Campylobacter* or *Salmonella* and *Campylobacter* and *Salmonella* prevalence included in the systematic review by year of when sampling and testing began.

Years	Campylobacter	Salmonella	Campylobacter and Salmonella
1951–1959	0	4	0
1960–1969	0	6	0
1970–1979	0	3	0
1980–1989	0	2	1
1990–1999	1	8	0
2000–2009	24	41	3
2010–2016	10	34	0
Unspecified	25	76	9
TOTAL	60	174	13

**Table 3:** Studies included in systematic review of the prevalence of *Campylobacter* and *Salmonella* in African food animals and animal food products by region, country, and animal species, 1953–2016.

Pathogen	Region	Country	No. of studies	Animal type investigated	References
Campylobacter	Central Africa	Cameroon	2	Chicken	(Garin et al., 2012, Nzouankeu et al., 2010)
		Democratic Republic of Congo	1	Goat	(a Mpalang et al., 2014)
		TOTAL	3		
	Eastern Africa	Ethiopia	6	Cattle, Goat, Pig, Poultry, Sheep	(Chanyalew et al., 2013, Dadi and Asrat, 2008, Ewnetu and Mihret, 2010, Kassa et al., 2007, Nigatu et al., 2015, Woldemariam et al., 2009)
		Kenya	2	Cattle, Goat, Pig, Poultry, Sheep	(Osano and Arimi, 1999, Turkson et al., 1988)
		Madagascar	1	Poultry	(Garin et al., 2012)
		Mozambique	1	Cattle	(Acha et al., 2004)
		Tanzania	11	Camel, Cattle, Goat, Pig, Poultry, Sheep	(Jacob et al., 2011, Jiwa et al., 1994, Kashoma et al., 2016, Kashoma et al., 2015, Komba et al., 2014, Kusiluka et al., 2005, Mdegela et al., 2011, Mdegela et al., 2007, Mdegela et al., 2006, Nonga and Muhairwa, 2010, Nonga et al., 2010)
		TOTAL	21		
	Northern Africa	Algeria	1	Poultry	(Messad et al., 2014)
		Egypt	17	Buffalo, Cattle, Goat, Poultry, Sheep	(Abd el-Aziz et al., 2002, Ali and Ouf, 2005, Awadallah et al., 2014, El-Gamal Galal et al., 1992, El-Jakee et al., 2015, El-Shibiny et al., 2012, El-Tras et al., 2015, Hassanain, 2011, Khadr et al., 2006, Khalafalla, 1990, Khalafalla et al., 2015, Khalifa et al., 2013, Nassar and El-Ela, 2000, Omara et al., 2015, Rady et al., 2011, Ramadan et al., 2015, Samaha et al., 2012)
		TOTAL	18		
	Southern Africa	South Africa	11	Cattle, Goat, Pig, Poultry, Sheep	(Bester and Essack, 2012, Cooper et al., 2001, Jonker and Picard, 2010, Mabote et al., 2011, Montwedi and Ateba, 2012, Richardson and Koornhof, 1979, Uaboi-Egbenni et al., 2010, Uaboi-Egbenni et al., 2011a, Uaboi-Egbenni et al., 2011b, Uaboi-Egbenni et al., 2012, van Nierop et al., 2005)
		TOTAL	11		
	Western Africa	Cote d'Ivoire	2	Poultry	(Gblossi Bernadette et al., 2012, Goualie et al., 2007)
		Ghana	2	Goat, Pig, Poultry	(Abrahams et al., 1990, Sackey et al., 2001)
		Nigeria	14	Cattle, Goat, Pig, Poultry, Sheep	(Adekeye et al., 1989, Akwuobu et al., 2010, Elegbe, 1983, Elegbe et al., 1987, Ngulukun et al., 2011, Ngulukun et al., 2010, Ofukwu et al., 2008, Okunlade et al., 2015, Olubunmi and Adeniran, 1986, Raji et al., 2000, Salihu et al., 2009a, Salihu et al., 2012, Salihu et al., 2009b, Uaboi-Egbenni et al., 2008)
		Senegal	4	Poultry	(Cardinale et al., 2003a, Cardinale et al., 2003b, Cardinale et al., 2006, Garin et al., 2012)
		TOTAL	22		
Salmonella	Central Africa	Cameroon	2	Cattle, Pig, Poultry	(Akoachere et al., 2009, Nzouankeu et al., 2010)
		Democratic Republic of Congo	1	Cattle, Goat, Pig, Poultry, Sheep	(Mahangaiko et al., 2015)
		TOTAL	3		
	Eastern Africa	Ethiopia	28	Camel, Cattle, Goat, Pig, Poultry, Sheep	(Abebe and Tafese, 2014, Addis et al., 2011, Alemayehu et al., 2003, Alemu and Zewde, 2012, Aragaw et al., 2007, Aragaw et al., 2010, Ashenafi, 1994, Bekele and Ashenafi, 2010, Beshatu et al., 2015, Dabassa, 2013, Dabassa and Bacha, 2012, Eguale et al., 2016, Ejeta et al., 2004, Garedew et al., 2015, Gebeyehu et al., 2013, Molla et al., 2006a, Molla et al., 1999, ,[Bibr bb1025][Bibr bb1040], Molla et al., 2006b, Muluneh and Kibret, 2015, Nyeleti et al., 2000, Sibhat et al., 2011, Teklu and Negussie, 2011, Tibaijuka et al., 2002, Woldemariam et al., 2005, Yizengaw, 2015, Molla et al., 2003)
		Kenya	3	Cattle, Pig, Poultry	(Gitter and Brand, 1970, Kikuvi et al., 2007, Wesonga et al., 2010)
		Mauritius	1	Poultry	(Phagoo and Neetoo, 2015)
		Mozambique	1	Cattle	(Acha et al., 2004)
		Rhodesia	1	Cattle	(Chambers, 1977)
		Rwanda	1	Cattle	(Niyonzima et al., 2016)
		Tanzania	4	Cattle, Goat, Poultry	(Hummel, 1974, Kusiluka et al., 2005, Mdegela et al., 2000, Otaru et al., 1990)
		Uganda	1	Pig	(Ikwap et al., 2014)
		Zambia	4	Cattle, Pig, Poultry	(Hang'ombe et al., 1999, Isogai et al., 2005, Kuroda et al., 2013, Ngoma et al., 1996)
		Zimbabwe	1	Poultry	(Gopo and Banda, 1997)
		TOTAL	45		
	Northern Africa	Algeria	5	Cattle, Poultry, Sheep	(Akam et al., 2004, Ammar et al., 2010, Guergueb et al., 2014, Mezali and Hamdi, 2012, Nouichi and Hamdi, 2009)
		Egypt	59	Buffalo, Camel, Cattle, Goat, Pig, Poultry, Sheep	(Abd el-Aziz et al., 2002, Abd El-Ghany et al., 2012, Abd-Elghany et al., 2015, Abdel-Maksoud et al., 2015, Abou El Hassan, 1996, ,[Bibr bb0095][Bibr bb0105], Ahmed et al., 2014, Al-Hazmi et al., 2013, Ali and Ouf, 2005, Aliaa et al., 2014, Amal et al., 2014, Amin and El-Rahman, 2015, Ammar et al., 2009, Eisa and Mohamed, 2004, Eissa et al., 2014, El-Gamal and El-Bahi, 2016, El-Naker et al., 2008, El-Tras et al., 2010, Elmossalami and Yassein, 1994, Farrag et al., 1954, Farrag and El-Afify, 1956, Farrag et al., 1962, Floyd et al., 1953, Gharieb et al., 2015, Ghoneim et al., 2015, Hamada et al., 1963, Hassb-Elnaby et al., 2011, Ibrahim, 2016, Khalafalla et al., 2015, Lotfi and Kamel, 1964b, Mahmoud and Hamouda, 2006, Mira and Eskandar, 2007, Moawad et al., 2013, Mohamed et al., 2014, Mohamed and Dapgh, 2007, Mousa et al., 1993, Moussa et al., 2013, Moussa et al., 2014, Nabawy et al., 2016, Nassar and El-Ela, 2000, Nawar and Khedr, 2014, Nossair et al., 2015, Osman et al., 2014a, Osman et al., 2014b, Osman et al., 2014c, Osman et al., 2010, Ouf, 2004, Rady et al., 2011, Randa et al., 2014, Refai et al., 1984, Sallam et al., 2014, Samaha et al., 2011, Samaha et al., 2012, Scharawe et al., 2009, Shaltout and Abdel-Aziz, 2004, Zahran and El-Behiry, 2014, Samaha et al., 2002, Lotfi and Kamel, 1964a)
		Morocco	6	Cattle, Poultry, Sheep	(Abdellah et al., 2008, Amara et al., 1994, Bouchrif et al., 2009, Cohen et al., 2007, Cohen et al., 2006, Khallaf et al., 2014)
		Sudan	7	Camel, Cattle, Goat, Poultry, Sheep	(Hag Elsafi et al., 2009, Ibrahim, 1974, Khan, 1970a, Khan, 1970c, Khan, 1970b, Mohammed et al., 2003, Yagoub and Mohamed, 1987)
		Tunisia	2	Cattle, Poultry, Sheep	(Abbassi-Ghozzi et al., 2012, Oueslati et al., 2016)
		TOTAL	78		
	Southern Africa	Botswana	5	Cattle, Poultry	(Gaedirelwe and Sebunya, 2008, Gashe and Mpuchane, 2000, Kapondorah and Sebunya, 2007, Miller, 1971, Motsoela et al., 2002)
		Namibia	1	Cattle	(Shilangale et al., 2015)
		South Africa	10	Cattle, Goat, Pig, Poultry, Sheep	(Iwu et al., 2016, Madoroba et al., 2016, Mathole et al., 2017, Nyamakwere et al., 2016, Prior and Badenhorst, 1974, Richardson et al., 1968, Tanih et al., 2015, van Nierop et al., 2005, Zishiri et al., 2016, Meara et al., 1977)
		TOTAL	16		
	Western Africa	Benin	2	Pig, Poultry	(Boko et al., 2013, Kpodekon et al., 2013)
		Burkina Faso	4	Cattle, Pig, Poultry, Sheep	(Bawa et al., 2015, Kagambèga et al., 2012, Kagambèga et al., 2011, Kagambèga et al., 2013)
		Cote d'Ivoire	1	Poultry	(Rene et al., 2014)
		Gambia, The	1	Goat, Poultry, Sheep	(Dione et al., 2011)
		Ghana	2	Cattle, Poultry	(Hughes et al., 2015, Sackey et al., 2001)
		Nigeria	30	Camel, Cattle, Goat, Pig, Poultry, Sheep	(Adesiji et al., 2011, Adesiyun and Oni, 1989, Adeyanju and Ishola, 2014, Adu-Gyamfi et al., 2012, Ajayi and Egbebi, 2011, Alao et al., 2012, Bata et al., 2016, Collard and Sen, 1956, Daniyan, 2011, Elegbe, 1983, Falade and Ehizokhale, 1981, Fashae et al., 2010, Iroha et al., 2011, Jajere et al., 2015, Kwaga, 1985, Nwachukwu et al., 2010, Oboegbulem and Muogbo, 1981, Ola Ojo, 1974, Olatoye, 2011, Olayemi et al., 1979, Oluyege and Ojo-Bola, 2015, Onyekaba and Njoku, 1986, Orji et al., 2005, Raufu et al., 2013, Raufu et al., 2009, Sen and Collard, 1957b, Sen and Collard, 1957a, Smith et al., 2016, Smith et al., 2009, Tafida et al., 2013)
		Senegal	5	Cattle, Poultry	(Bada-Alambedji et al., 2006, Cardinale et al., 2003b, Dione et al., 2009, Missohou et al., 2009, Stevens et al., 2006)
		TOTAL	45		

### 3.1 Campylobacter

**Fig. 3:**
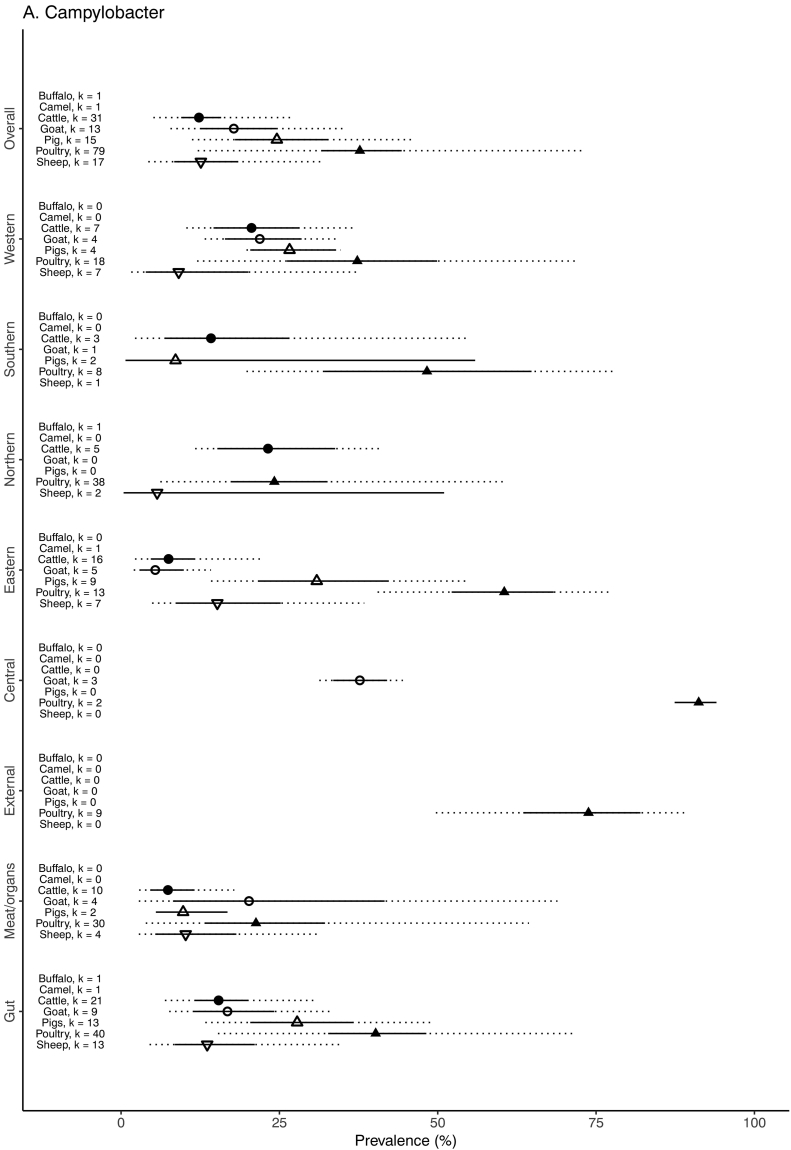
Forest plot with adjusted prevalence estimates for *Campylobacter* in food animals and meat for each animal species, sample type and African region, 1953–2016. 95% confidence intervals shown in solid line. 80% prediction intervals shown with dotted line. Adjusted prevalence not estimated when number of studies (k) < 2, 1979–2015.

**Fig. 4:**
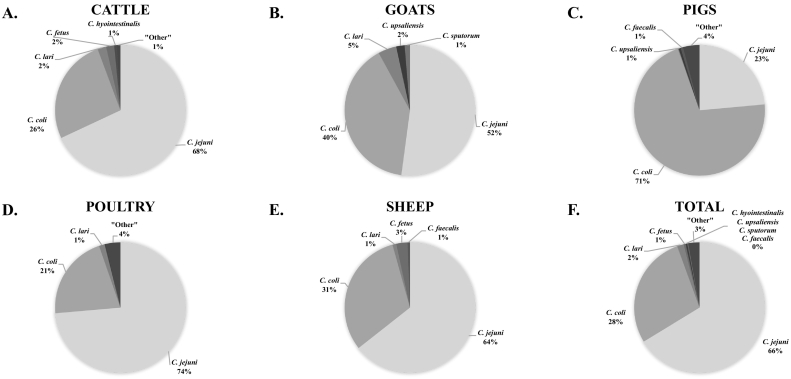
Pie graphs showing the breakdown of *Campylobacter* species reported when typed from (A) Cattle, (B) Goats, (C) Pigs, (D), Poultry, (E) Sheep and (F) all African food animal species combined, 1953–2016.

The unadjusted prevalence of *Campylobacter* by animal species among various sample types for each African region by source animal species is shown in Table 4. The weighted prevalence of *Campylobacter* by animal species, sample type, and geographic region is summarized in Fig. 3. The prevalence was highest in poultry samples (37.7%, 95% CI 31.6–44.3, 80% PI 12.1–72.7) followed by pig samples (24.6%, 95% CI 18.0–32.7, 80% PI 11.2–45.9), goat samples (17.8%, 95% CI 12.6–24.5, 80% PI 7.8–35.6), sheep samples (12.6%, 95% CI 8.4–18.5, 80% PI 4.3–31.7), and cattle samples (12.3%, 95% CI 9.5–15.8, 80% PI 5.1–26.7). One study reported attempted isolation or detection of *Campylobacter* from camels and buffalo, with zero positive cases for both species. Fig. 4 shows the breakdown of *Campylobacter* species between food animals. For all animal samples, *Campylobacter* were significantly less likely to be isolated or detected from meat or organ samples than from gut samples (OR = 0.67, 95% CI 0.56–0.80), and significantly more likely to be isolated or detected from external samples than gut samples (OR = 1.77, 95% CI 1.19–2.65) (Table 5). With adjustment for sample and species type, the odds of contamination of food animal samples with *Campylobacter* from Central Africa was significantly higher than the referent region of Northern Africa (OR = 9.06, 95% CI 1.92–42.70). The odds of contamination were significantly lower in samples from pigs (OR = 0.60, 95% CI 0.49–0.73) and all other species (OR = 0.29, 95% CI 0.25–0.33) when compared to samples from poultry. There was evidence that the inclusion of region, sample and species type as fixed effects improved model fit (Χ^2^ = 10.6, *p* = 0.03; Χ^2^ = 32.8, *p* ≤0.001; Χ^2^ = 332.7, p ≤0.001, respectively).

**Table 4:** Combined unadjusted *Campylobacter* prevalence and species for food animal type and various sample types, 1979–2015.

Host animal type	Sample stage	Sample type	No. of samples tested	No. of samples positive (%)		*Campylobacter* species when isolates were typed from positive samples					
						C. jejuni (%)		C. coli (%)		Other *C.* spp.^a^ (%)	
Buffalo	Gut	Intestinal contents	55	0	(0.0)	–	–	–	–	–	–
		TOTAL	55	0	(0.0)	–	–	–	–	–	–
Camel	Gut	Faeces/Rectal swab	3	0	(0.0)	–	–	–	–	–	–
		TOTAL	3	0	(0.0)	–	–	–	–	–	–
Cattle	Gut	Faeces/Rectal swab	4957	733	(14.8)	512	(68.7)	187	(25.1)	46	(6.2)
	Gut	Intestinal contents	80	4	(5.0)	NT	–	NT	–	NT	–
	Meat/organ	Carcass	827	55	(6.7)	37	(67.3)	16	(29.1)	2	(3.6)
	Meat/organ	Gallbladder	100	12	(12.0)	0	(0.0)	12	(100)	0	(0.0)
	Meat/organ	Liver	30	8	(26.7)	5	(62.5)	3	(37.5)	0	(0.0)
	Meat/organ	Meat	521	25	(4.8)	21	(84.0)	4	(16.0)	0	(0.0)
	Meat/organ	Tripe	30	1	(3.3)	NT	–	NT	–	NT	–
		TOTAL	6545	838	(12.8)	575	(68.0)	222	(26.3)	48	(5.7)
Goat	Gut	Faeces/Rectal swab	2372	472	(19.9)	283	(59.0)	149	(31.0)	48	(10.0)
	Meat/organ	Carcass	180	17	(9.4)	12	(70.6)	5	(29.4)	0	(0.0)
	Meat/organ	Meat	269	80	(29.7)	17	(20.7)	65	(79.3)	0	(0.0)
	Meat/organ	Stomachs	86	32	(91.3)	7	(21.9)	25	(78.1)	0	(0.0)
		TOTAL	2907	601	(20.7)	319	(53.1)	244	(40.6)	48	(8.0)
Pigs	Gut	Caeca/Intestine	454	59	(13.0)	36	(61.0)	23	(39.0)	0	(0.0)
	Gut	Faeces/Rectal swab	1408	467	(33.2)	83	(17.8)	354	(76.1)	28	(6.0)
	Meat/organ	Carcass	66	7	(10.6)	6	(85.7)	1	(14.3)	0	(0.0)
	Meat/organ	Meat	47	4	(8.5)	1	(25.0)	2	(50.0)	1	(25.0)
		TOTAL	1975	537	(27.2)	126	(23.6)	380	(71.0)	29	(5.4)
Poultry	Gut	Caeca	1902	759	(39.9)	442	(71.9)	161	(26.2)	12	(2.0)
	Gut	Faeces/Cloaca swab	5548	2457	(44.3)	1893	(82.6)	276	(12.0)	122	(5.3)
	Meat/organ	Carcass	653	335	(51.3)	130	(55.3)	97	(41.3)	8	(3.4)
	Meat/organ	Gallbladder or bile	65	3	(4.6)	3	(100)	0	(0.0)	0	(0.0)
	Meat/organ	Giblet	37	15	(40.5)	5	(100)	0	(0.0)	0	(0.0)
	Meat/organ	Gizzard	250	43	(17.2)	43	(100)	0	(0.0)	0	(0.0)
	Meat/organ	Heart	250	11	(4.4)	11	(100)	0	(0.0)	0	(0.0)
	Meat/organ	Kidney	25	2	(8.0)	2	(100)	0	(0.0)	0	(0.0)
	Meat/organ	Liver	300	75	(25.0)	68	(100)	0	(0.0)	0	(0.0)
	Meat/organ	Meat	1193	257	(21.5)	132	(71.4)	10	(5.4)	43	(23.2)
	Meat/organ	Spleen	200	17	(8.5)	17	(100)	0	(0.0)	0	(0.0)
	External	Skin	1405	948	(67.5)	346	(47.7)	341	(47.0)	38	(5.2)
		TOTAL	11,828	4922	(41.6)	3092	(73.6)	885	(21.1)	223	(5.3)
Sheep	Gut	Faeces/Rectal swab	1430	248	(17.3)	148	(59.7)	87	(35.1)	13	(5.2)
	Gut	Intestinal contents	300	17	(5.7)	11	(64.7)	4	(23.5)	2	(11.8)
	Meat/organ	Carcass	288	38	(13.2)	31	(81.6)	7	(18.4)	0	(0.0)
	Meat/organ	Gallbladder	250	10	(4.0)	8	(80.0)	1	(10.0)	1	(10.0)
	Meat/organ	Meat	114	12	(10.5)	10	(83.3)	2	(16.7)	0	(0.0)
		TOTAL	2382	325	(13.6)	208	(64.0)	101	(31.1)	16	(4.9)

a Where “Other” Campylobacter species include: C. faecalis, C. fetus, C. hyointesinalis, C. lari, C. sputorum, C. upsaliensis.

**Table 5:** Odds Ratios (OR) with 95% Confidence intervals (CI) for fixed effects from multi-level multivariable logistic regression models for *Campylobacter* and *Salmonella* prevalence for sample types, 1953–2016.

Model	Fixed effects	Campylobacter		Salmonella	
		OR	95% CI	OR	95% CI
All	Region				
	Northern	Baseline		Baseline	
	Central	9.06	(1.92–42.70)⁎	6.64	(1.37–32.24)⁎
	Eastern	1.23	(0.61–2.46)	1.36	(0.91–2.05)
	Southern	2.08	(0.81–5.39)	1.35	(0.69–2.68)
	Western	1.59	(0.79–3.21)	1.79	(1.10–2.92)⁎
	Sample type				
	Gut	Baseline		Baseline	
	External	1.77	(1.19–2.65)⁎	1.77	(1.44–2.18)⁎
	Internal	0.67	(0.56–0.80)⁎	0.86	(0.79–0.95)⁎
	Species type				
	Poultry	Baseline		Baseline	
	Pigs	0.60	(0.49–0.73)⁎	0.82	(0.63–1.07)
	All other	0.29	(0.25–0.33)⁎	0.47	(0.42–0.54)⁎
Cattle	Region				
	Northern	Baseline		Baseline	
	Central	–		6.53	(0.66–64.87)
	Eastern	0.60	(0.19–1.86)	1.07	(0.53–2.17)
	Southern	0.96	(0.19–4.76)	0.57	(0.18–1.83)
	Western	1.45	(0.42–5.02)	1.88	(0.77–4.61)
	Sample type				
	Gut	Baseline		Baseline	
	External	–		8.11	(5.5–11.96)⁎
	Internal	0.40	(0.26–0.61)⁎	0.97	(0.81–1.17)
Goats	Region				
	Northern	–	–	Baseline	
	Central	10.3	(7.0–15.1)⁎	17.3	(0.65–461.6)
	Eastern	Baseline		4.8	(0.74–31.8)
	Southern	8.7	(5.3–14.1)⁎	0.45	(0.02–12.8)
	Western	5.5	(3.7–8.1)⁎	3.4	(0.5–22.5)
	Sample type				
	Gut	Baseline		Baseline	
	External	–	–	0.91	(0.23–3.6)
	Internal	1.5	(1.1–2.1)⁎	1.3	(0.68–2.6)
Sheep	Region				
	Northern	Baseline		Baseline	
	Central	–	–	6.5	(1.4–30.3)⁎
	Eastern	2.4	(0.42–13.8)	1.1	(0.55–2.2)
	Southern	7.1	(0.63–79.5)	0.25	(0.07–0.98)⁎
	Western	2.2	(0.35–13.6)	3.1	(1.3–7.1)⁎
	Sample type				
	Gut	Baseline		Baseline	
	External	–	–	1.4	(0.93–2.0)
	Internal	1.27	(0.78–2.1)	0.85	(0.4–2.1)
Pigs	Region				
	Northern	–		Baseline	
	Central	–		10.92	(1.36–87.54)⁎
	Eastern	Baseline		2.67	(0.53–13.44)
	Southern	0.17	(0.06–0.52)⁎	2.56	(0.39–16.70)
	Western	0.42	(0.16–1.15)	3.46	(0.67–18.03)
	Sample type				
	Gut	Baseline		Baseline	
	External	–		–	
	Internal	0.19	(0.09–0.40)⁎	1.07	(0.83–1.39)
Poultry	Region				
	Northern	Baseline		Baseline	
	Central	13.3	(4.35–40.74)⁎	3.53	(0.42–29.93)
	Eastern	1.87	(0.73–4.83)	1.12	(0.45–2.81)
	Southern	2.89	(0.78–10.68)	2.11	(0.63–7.06)
	Western	1.45	(0.56–3.71)	1.81	(0.86–3.80)
	Sample type				
	Gut	Baseline		Baseline	
	External	1.57	(1.03–2.39)⁎	0.73	(0.55–0.98)⁎
	Internal	0.55	(0.41–0.73)⁎	0.45	(0.39–0.53)⁎

⁎ *p* < 0.05.

Between study heterogeneity on the basis of the I^2^ statistic was high (>90%) for all livestock species, except cattle for which a relatively small proportion of the between study variation (15.8%) was estimated to be due to prevalence differences rather than chance variation. This is supported by values for MOR, which suggest that when comparing pairs of samples from two randomly selected studies, the odds of *Campylobacter* sample contamination in the study reporting higher prevalence would, in median, be 3.2 times the odds of sample contamination in the lower prevalence study, with control for region, sample type, and species type. For the species-specific models, between study heterogeneity on the basis of MOR was notably high for poultry, in which the estimated median difference in odds of sample contamination between a low and high prevalence study was 4.1 (Table 6).

**Table 6:** Inter-study heterogeneity based on the I^2^ statistic and median odds ratio (MOR) for *Campylobacter* and *Salmonella* for all included studies (overall) and for included studies for each African food animal species for included studies, 1953–2016.

	Campylobacter		Salmonella	
	I^2^	MOR	I^2^	MOR
Overall	96.7%	3.2	98.3%	3.7
Camels	–	–	72.9%	3.0
Cattle	15.8%	2.2	97.1%	4.5
Goats	92.1%	n.d.	80.6%	3.6
Pigs	90.4%	1.8	93.5%	2.7
Poultry	96.9%	4.1	95.3%	4.0
Sheep	90.8%	2.4	77.6%	1.7

#### 3.1.1 Poultry

The adjusted prevalence on the basis of 7450 poultry gut samples was 40.2% (95% CI 32.7–48.2), 73.8% (95% CI 63.5–82.0) in 1405 poultry external samples, and 21.3% (95% CI 13.3–32.2), among 2973 meat or organ samples (Fig. 3). On the basis of the multivariable model, *Campylobacter* were significantly less likely to be isolated or detected in meat or organ samples than gut samples (OR = 0.55, 95% CI 0.41–0.73), but significantly more likely to be isolated or detected in external samples than gut samples (OR = 1.57, 95% CI 1.03–2.39) (Table 5). The adjusted *Campylobacter* prevalence was highest in Central Africa (91.2, 95% CI 87.4–94.0) and lowest in Northern Africa (24.2%, 95% CI 17.3–32.6) (Fig. 3). On the basis of the multivariable model, *Campylobacter* were significantly more likely to be isolated or detected in poultry samples from Central Africa than the referent region of Northern Africa (OR = 13.3, 95% CI 4.35–40.74) (Table 5). However, there was no evidence that the inclusion of region as a fixed effect improved model fit (Χ^2^ = 7.4, *p* = 0.11). There was strong evidence for an improvement in model fit with the inclusion of sample type (Χ^2^ = 28.1, *p* ≤0.001).

Among the 4922 poultry samples from which *Campylobacter* was isolated or detected, 4200 (85.7%) isolates were speciated. Of 4200 speciated isolates, 3092 (73.6%) were *C. jejuni*, 885 (21.1%) were *C. coli*, 152 (3.6%) were reported as ‘other’, 56 (1.3%) were *C. lari*, 10 (0.2%) were *C. upsaliensis*, and five (0.1%) were *C. fetus* (Table 4).

#### 3.1.2 Pigs

The adjusted prevalence on the basis of 1862 pig gut samples was 27.8% (95% CI 20.4–36.7). No pig external samples were tested for *Campylobacter*. Among 113 pig meat or organ samples, the adjusted prevalence was 9.8% (95% CI 5.5–16.8) (Fig. 3). *Campylobacter* were significantly less likely to be isolated or detected from pig meat or organ samples than from pig gut samples (OR = 0.19, 95% CI 0.09–0.40) (Table 5). The adjusted *Campylobacter* prevalence was 30.9% (95% CI 21.6–42.1) for samples from Eastern Africa, 26.6% (95% CI 20.4–33.8) for samples from Western Africa, and 8.6% (95% CI 0.7–55.9) for samples from Southern Africa (Fig. 3). No studies from Central or Northern Africa reported on *Campylobacter* prevalence in pigs. When adjusted for sample type, the prevalence of *Campylobacter* in samples from Southern Africa was significantly lower (OR = 0.17, 95% CI 0.06–1.15) than the referent region of Eastern Africa (Table 5). There was evidence that the inclusion of region and sample type improved model fit (Χ^2^ = 7.7, *p* = 0.02; Χ^2^ = 24.0, *p* ≤0.001, respectively).

Among the 537 pig samples from which *Campylobacter* was isolated or detected, 535 isolates (99.6%) were speciated. Of 535 speciated isolates, 126 (23.6%) were *C. jejuni*, 380 (71.1%) were *C. coli*, 22 (4.1%) were reported as ‘other’, three (0.6%) were *C. hyointestinalis*, three (0.6%) were *C. faecalis*, and one (0.2%) was *C. lari* (Table 4).

#### 3.1.3 Goats

The adjusted *Campylobacter* prevalence on the basis of 2372 goat gut samples was 16.8% (95% CI 11.4–24.6). No goat external samples were tested. Among 535 goat meat or organ samples, the adjusted prevalence was 20.2% (95% CI 8.2–41.6) (Fig. 3) After controlling for region, goat meat or organ samples were significantly more likely to be contaminated than gut samples (OR = 1.5, 95% CI 1.1–2.1) (Table 5). The adjusted prevalence of *Campylobacter* contamination was highest in the Central region (37.7%, 95% CI 33.5–42.0) and lowest in the Eastern region (5.4%, 95% CI 2.9–9.9) (Fig. 3). No samples were collected from the Northern region. There were significant differences in the odds of *Campylobacter* contamination when comparing samples from Central, Southern and Western regions with the Eastern baseline (Table 5). There was evidence that the inclusion of region and sample type as fixed effects improved model fit (Χ^2^ = 23.3, *p* ≤0.001; Χ^2^ = 6.3, *p* = 0.01, respectively).

For *Campylobacter* from goats, 616 isolates were speciated from 601 samples. Of these 616 isolates, 321 (52.1%) were *C. jejuni*, 246 (39.9%) were *C. coli*, 28 (4.5%) were *C. lari,* 13 (2.1%) were *C. upsaliensis*, and eight (1.3%) were *C. sputorum* (Table 4).

#### 3.1.4 Sheep

The adjusted prevalence of *Campylobacter* contamination in 1730 sheep gut samples was 13.6%, (95% CI 8.5–21.1) and 10.2% (95% CI 5.4–18.2) on the basis of 652 meat or organ samples (Fig. 3). No external samples were reported. There was no evidence of a difference between the odds of contamination between sample types in the multivariable regression (Table 5). The adjusted prevalence of *Campylobacter* contamination was highest in the Southern region (30.0%, 95% CI 25.1–35.4) and lowest in the Northern region (5.7%, 95% CI 0.4–51.0) (Fig. 3). There was no evidence that including region or sample type improved model fit (Χ^2^ = 2.5, *p* = 0.48; Χ^2^ = 0.9, *p* = 0.35, respectively).

For *Campylobacter* from sheep, 323 isolates were speciated. Of these 323 isolates, 208 (64.4%) were *C. jejuni*, 101 (31.3%) were *C. coli*, nine (2.8%) were *C. fetus*, three (0.9%) were *C. lari,* and two (0.6%) were *C. faecalis* (Table 4).

#### 3.1.5 Cattle

The adjusted prevalence derived from 5037 cattle gut samples was 15.4% (95% CI 11.7–20.0, 80% PI 6.9–30.8) compared to 7.4% (95% CI 4.6–11.6, 80% PI 2.9–17.9) from 1508 meat or organ samples (Fig. 3). No cattle external samples were tested for *Campylobacter*. On the basis of the cattle-specific multivariable model, *Campylobacter* were significantly less likely to be isolated or detected from meat or organ samples than from gut samples (OR = 0.40, 95% CI 0.26–0.61) (Table 5). The adjusted prevalence of contamination was highest in Northern Africa (23.2%, 95% CI 15.2–33.8) and lowest in Eastern Africa (7.5%, 95% CI 4.7–11.7) (Fig. 3), but there was no evidence of a difference in the odds of *Campylobacter* contamination between samples from Northern Africa and any other region from the multivariable model (Table 5). No studies from Central Africa reported on *Campylobacter* prevalence in cattle samples. There was evidence that the inclusion of sample type as a fixed effect improved model fit (Χ^2^ = 18.6, *p* ≤0.001), but no evidence for geographic region (Χ^2^ = 4.0, *p* = 0.26).

For *Campylobacter* from cattle, 845 isolates were speciated. Of these 845 isolates, 575 (68.0%) were *C. jejuni*, 222 (26.3%) were *C. coli*, 18 (2.1%) were *C. lari*, 17 (2.0%) were *C. fetus*, eight (0.9%) were reported as ‘other’, and five (0.6%) were *C. hyointestinalis* (Table 4).

### 3.2 Salmonella

**Fig. 5:**
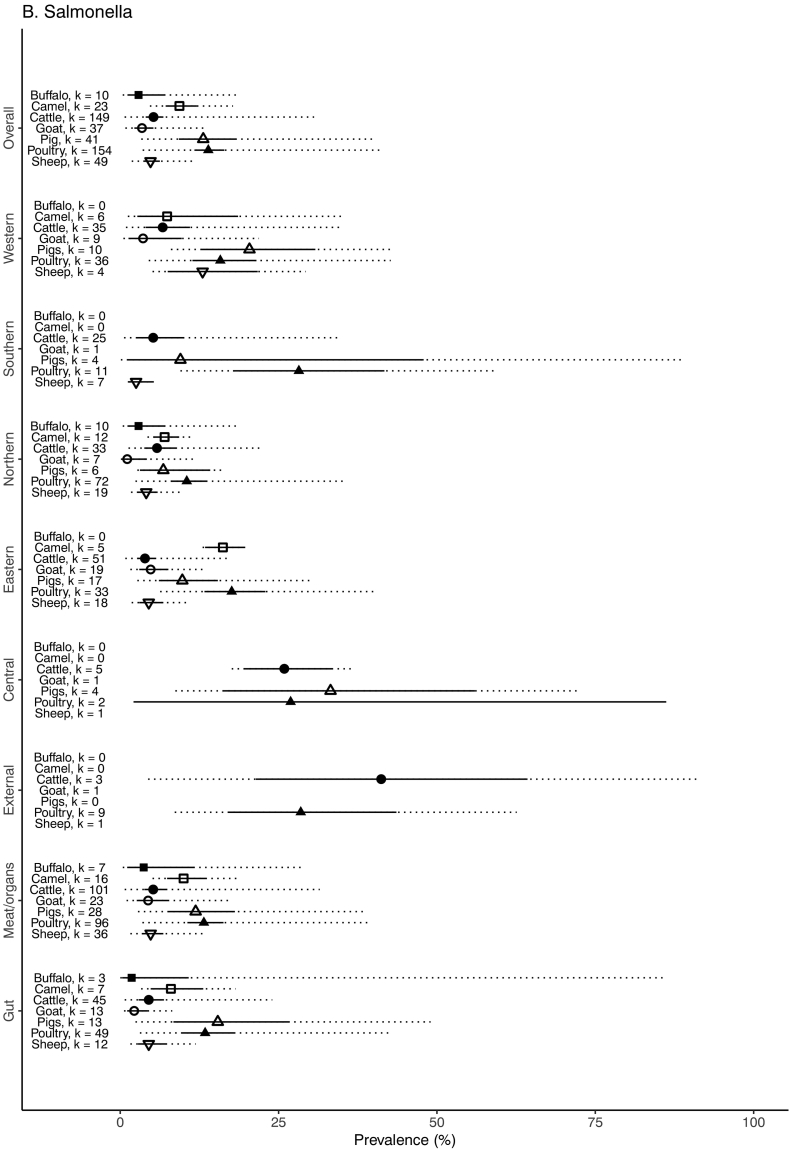
Forest plot with adjusted prevalence estimates for *Salmonella* in food animals and meat for each animal species, sample type and African region, 1953–2016. 95% confidence intervals shown in solid line. 80% prediction intervals shown with dotted line. Adjusted prevalence not estimated when number of studies (k) < 2, 1953–2016.

The unadjusted prevalence of *Salmonella* and serovar composition in the various sample types for each African region for each host animal species is shown in Table 7. The weighted prevalence of *Salmonella* by animal species, sample type, and geographic region is summarized in Fig. 5. The prevalence of *Salmonella* was highest in poultry samples (13.9% (95% CI 11.7–16.4, 80% PI 3.5–41.5)), followed by pig samples (13.1% (95% CI 9.3–18.3, 80% PI 3.3–39.9)), camel samples (9.3% (95% CI 7.2–12.1, 80% PI 4.7–17.8)), cattle samples (5.3% (95% CI 4.0–6.8, 80% PI 4.0–6.8)), sheep samples (4.8% (95% CI 3.6–6.3, 80% PI 1.8–12.0)), goat samples (3.4% (95% CI 2.2–5.2, 80% PI 2.2–5.2)) and buffalo samples (2.9% (95% 1.1–7.1, 80% PI 1.1–7.1)). *Salmonella* were significantly more likely to be detected in samples from Central and Western Africa compared to the referent region of North Africa (OR = 6.64, 95% CI 1.37–32.24 and OR = 1.79, 95% CI 1.01–2.92, respectively). Samples from pigs and all other species were less likely to be found to be contaminated than samples from poultry, but this was only significant in the case of the all other species category (OR = 0.47, 95% CI 0.42–0.54) (Table 5). There was weak evidence that the inclusion of region improved model fit (Χ^2^ = 8.9, *p* = 0.06) with stronger evidence for an improvement with the inclusion of sample type (Χ^2^ = 48.9, *p* ≤0.001) and species type (Χ^2^ = 134.2, p ≤0.001). Supplementary Table 2 details all reported *Salmonella* serovars quantified in each animal species by region.

Between study heterogeneity was high for all studies, and above 90% for cattle, pigs, and poultry. Values of MOR suggest that a sample collected in a higher prevalence study would have, in median, around 3.7 times the odds of *Salmonella* sample contamination than a sample collected from a lower prevalence study, with control for region, sample type, and species type. For the species-specific models, between study heterogeneity on the basis of MOR was notably high for cattle and poultry, in which the estimated median difference in odds of sample contamination between a low and high prevalence study was 4.5 and 4.0, respectively (Table 6).

**Table 7:** Combined unadjusted *Salmonella* prevalence and three most numerous serovars for food animal type and various sample types, 1953–2016.

Animal species	Sample stage	Sample type	No. of samples tested	No. of samples positive (%)		Number of isolates typed to serovar^a^ (%)		Ranking of most commonly typed *Salmonella enterica* serovars					
								1		2		3	
								Serovar name	No. (%)	Serovar name	No. (%)	Serovar name	No. (%)
Buffalo	Gut	Faeces/Rectal swab	2237	53	(2.4)								
	Meat/organ	Bile	900	12	(1.3)								
	Meat/organ	Lymph node	999	8	(0.8)								
	Meat/organ	Meat	88	10	(11.4)								
		TOTAL	4224	83	(2.0)	83	(100)	Typhimurium	35 (42.2)	Dublin	13 (15.7)	Anatum	8 (9.6)
Camel	Gut	Faeces/Rectal swab	811	33	(4.1)								
	Gut	Intestine	450	32	(7.1)								
	Meat/organ	Liver	269	20	(7.4)								
	Meat/organ	Lymph node	886	95	(10.7)								
	Meat/organ	Meat	403	54	(13.4)								
	Meat/organ	Spleen	169	24	(14.2)								
		TOTAL	2988	258	(8.6)	251	(97.3)	Saintpaul	49 (19.5)	Typhimurium	27 (10.8)	Branderup	26 (10.4)
Cattle	Gut	Faeces/Rectal swab	8683	517	(6.0)								
	Gut	Intestine	3090	143	(4.6)								
	Meat/organ	Carcass	6019	162	(2.7)								
	Meat/organ	Gallbladder/bile	6558	135	(2.1)								
	Meat/organ	Heart	37	1	(2.7)								
	Meat/organ	Kidney	126	6	(4.8)								
	Meat/organ	Liver	1248	40	(3.2)								
	Meat/organ	Lymph node	3835	139	(3.6)								
	Meat/organ	Meat	23,998	1050	(4.4)								
	Meat/organ	Meat rinse	4396	19	(0.4)								
	Meat/organ	Spleen	42	1	(2.4)								
	Meat/organ	Tongue	13,680	199	(1.5)								
	Meat/organ	Tripe	403	23	(5.7)								
	External	Hide	177	74	(41.8)								
		TOTAL	72,292	2509	(3.5)	1637	(65.2)	Typhimurium	289 (17.7)	Anatum	124 (7.6)	Enteritidis	120 (7.3)
Goat	Gut	Faeces/Rectal swab	1411	30	(2.1)								
	Gut	Intestine	685	5	(0.7)								
	Meat/organ	Carcass	309	49	(15.9)								
	Meat/organ	Gallbladder/bile	625	0	(0.0)								
	Meat/organ	Liver	160	3	(1.9)								
	Meat/organ	Lymph node	911	33	(3.6)								
	Meat/organ	Meat	583	24	(4.1)								
	Meat/organ	Spleen	160	2	(1.3)								
	External	Skin	60	3	(5.0)								
		TOTAL	4904	149	(3.0)	47	(32.4)	Typhimurium	18 (12.4)	Typhi	5 (3.4)	Amersfoort Poona Derby “Other”/UT	2 (1.4)
Pig	Gut	Caeca/Intestine	510	92	(18.0)								
	Gut	Faeces/Rectal swab	2074	372	(17.9)								
	Meat/organ	Carcass	371	22	(5.9)								
	Meat/organ	Gallbladder/bile	660	4	(0.6)								
	Meat/organ	Heart	1	0	(0.0)								
	Meat/organ	Kidney	4	0	(0.0)								
	Meat/organ	Liver	121	18	(14.9)								
	Meat/organ	Lymph node	1184	173	(14.6)								
	Meat/organ	Meat	425	51	(12.0)								
	Meat/organ	Tongue	117	30	(25.6)								
		TOTAL	5467	762	(13.9)	478	(62.7)	Hadar	85 (17.9)	Group O:2	48 (10.0)	Saintpaul	46 (9.6)
Poultry	Gut	Caeca/Intestine	1997	360	(18.0)								
	Gut	Faeces/Cloaca swab	10,060	1321	(13.1)								
	Meat/organ	Carcass	1752	335	(19.1)								
	Meat/organ	Gallbladder	279	19	(6.8)								
	Meat/organ	Giblets	42	18	(42.9)								
	Meat/organ	Gizzard	876	267	(30.5)								
	Meat/organ	Heart	452	38	(8.4)								
	Meat/organ	Kidney	90	0	(0.0)								
	Meat/organ	Liver	1852	180	(9.7)								
	Meat/organ	Meat	6578	712	(27.3)								
	Meat/organ	Spleen	425	20	(4.7)								
	External	Feathers	120	61	(50.8)								
	External	Skin	907	284	(31.3)								
		TOTAL	25,430	3615	(14.2)	2098	(58.0)	Enteritidis	464 (22.1)	Typhimurium	311 (14.8)	Typhi	174 (8.3)
Sheep	Gut	Faeces/Rectal swab	2370	97	(4.1)								
	Gut	Intestine	1797	28	(1.6)								
	Meat/organ	Carcass	381	25	(6.6)								
	Meat/organ	Gallbladder/bile	2305	31	(1.3)								
	Meat/organ	Heart	207	0	(0.0)								
	Meat/organ	Kidney	9	0	(0.0)								
	Meat/organ	Liver	190	4	(2.1)								
	Meat/organ	Lymph node	2817	71	(2.5)								
	Meat/organ	Meat	936	68	(7.3)								
	Meat/organ	Spleen	181	1	(0.6)								
	External	Skin	142	7	(4.9)								
		TOTAL	11,335	332	(2.9)	165	(49.7)	Typhimurium	68 (20.5)	Enteritidis	14 (4.2)	Eastbourne	11 (3.3)

UT = Untypeable.

a Serovars were excluded from number of isolates typed to serovar if numbers of isolates identified were not stated.

#### 3.2.1 Poultry

The adjusted prevalence on the basis of 12,057 poultry gut samples was 13.4% (95% CI 9.8–18.0), 28.5% (95% CI 17.1–43.6) on the basis of 1027 external samples, and 13.2% (95% CI 10.6–16.3) on the basis of 12,346 poultry meat or organ samples. *Salmonella* were significantly less likely to be isolated or detected from both external (OR = 0.73, 95% CI 0.55–0.98) and meat or organ samples (OR = 0.45, 95% CI 0.39–0.53) when compared with the poultry gut sample baseline (Table 5). The adjusted prevalence in poultry samples was highest in the Southern region (28.2%, 95% CI 17.8–41.7) and lowest in the Northern region (10.5%, 95% CI 8.0–13.6) (Fig. 5). There was no evidence for improvement in model fit with the inclusion of region (Χ^2^ = 4.1, *p* = 0.4), but strong evidence for improvement with the inclusion of sample type (Χ^2^ = 97.1, *p* ≤0.001).

In total, *Salmonella* was isolated or detected in 3615 poultry samples and 2236 (61.9%) were serotyped. Among the 2236 isolates serotyped from poultry samples, 464 (20.8%) were *Salmonella enterica* serovar Enteritidis, 311 (13.9%) were *Salmonella enterica* serovar Typhimurium, and 174 (7.8%) were *Salmonella enterica* serovar Typhi (Table 7).

#### 3.2.2 Pigs

The adjusted *Salmonella* prevalence on the basis of 2584 pig gut samples was 15.4% (95% CI 8.4–26.6). No pig external samples were tested for *Salmonella*. Among 2883 pig meat or organ samples, the adjusted prevalence was 11.9% (95% CI 7.6–18.1) (Fig. 5). There was no evidence for differences in the odds of *Salmonella* contamination when comparing pig gut and meat or organ samples. The adjusted *Salmonella* prevalence was highest in Central Africa (33.2% (95% CI 16.3–56.0)) and lowest in Northern Africa (6.8% (95% CI 3.1–14.2)). The odds of *Salmonella* contamination were significantly higher in the Central region than the Northern region baseline (OR = 10.9 (95% CI 1.36–87.5)) (Table 5). There was no evidence for improvement in model fit with the inclusion of either region (Χ^2^ = 4.8, *p* = 0.3) or sample type (Χ^2^ = 0.29, *p* = 0.59).

In total, *Salmonella* was isolated or detected in 762 pig samples and 428 (56.2%) of these were serotyped. Among the 494 isolates serotyped from pig samples, 85 (19.9%) were *Salmonella enterica* serovar Hadar, 46 (10.7%) were *Salmonella enterica* Saintpaul and 40 (9.3%) *Salmonella enterica* serovar Eastbourne (Table 7).

#### 3.2.3 Cattle

The adjusted *Salmonella* prevalence on the basis of 11,773 cattle gut samples was 4.5% (95% CI 2.9–6.9), 5.2% on the basis of 60,342 meat or organ samples, and 41.2% on the basis of 177 external samples (Fig. 5). *Salmonella* were significantly more likely to be isolated or detected from cattle external samples than cattle gut samples (OR = 8.11, 95% CI 5.5–11.96) (Table 5). The prevalence in cattle samples was highest in the Central region (25.9%, 95% CI 19.6–33.4) and lowest in the Eastern region (3.9%, 95% CI 2.7–5.5) (Fig. 5). There was no evidence for improvement in model fit with the inclusion of region (Χ^2^ = 6.0, *p* = 0.2), but strong evidence for improvement with the inclusion of sample type (Χ^2^ = 112.1, p ≤0.001).

In total, *Salmonella* was isolated or detected in 2509 cattle samples and 1750 (69.7%) of those were serotyped. Among the 1750 isolates serotyped from cattle, 289 (16.5%) were *Salmonella enterica* serovar Typhimurium, 124 (7.1%) were *Salmonella enterica* serovar Anatum, and 120 (6.9%) were *Salmonella enterica* serovar Enteritidis (Table 7).

#### 3.2.4 Sheep

The adjusted *Salmonella* prevalence on the basis of 4167 sheep gut samples was 4.5% (95% CI 2.8–7.2), and 4.8% (95% CI 3.4–6.8) on the basis of 7026 meat or organ samples (Fig. 5). A single study reported a prevalence of 4.9% for sheep external samples (*n* = 142). There was no evidence for a difference in the odds of contamination by sample type (Table 5). Like goats, the adjusted prevalence was highest in sheep samples from the Central region (25.0%, based on a single study) and lowest in the Southern region (2.5%, 95% CI 1.2–5.3) (Fig. 5). The odds of sample contamination were significantly elevated in Central (OR = 6.5, 95% CI 1.4–30.3) and Western regions (OR = 3.1, 95% CI 1.3–7.1), and significantly reduced in the Southern region (OR = 0.25, 95% CI 0.07–0.98) (Table 5). There was no evidence for improvement in model fit with the inclusion of sample type (Χ^2^ = 4.9, *p* = 0.29), but strong evidence for region (Χ^2^ = 14.4, *p* = 0.006).

In total, *Salmonella* was isolated or detected in 332 sheep samples and 165 (49.7%) of these were serotyped. Among the 165 isolates serotyped from sheep samples, 69 (41.8%) were *Salmonella enterica* serovar Typhimurium, 14 (8.5%) were *Salmonella enterica* Enteritidis and 11 (6.7%) *Salmonella enterica* serovar Eastbourne (Table 7).

#### 3.2.5 Goats

The adjusted *Salmonella* prevalence on the basis of 2096 goat gut samples was 2.2% (95% CI 1.1–4.3). Among 2748 goat meat or organ samples, the adjusted prevalence was 4.4% (95% CI 2.6–7.5) (Fig. 5). A single study reported on *Salmonella* detection in goat external samples. The adjusted prevalence was highest in the Central region (16.7%) and lowest in the Southern region (0.4%) (both based on a single study). There was no evidence of a difference in the odds of contamination by sample type or geographic region on the basis of a multivariable model (Table 3, Table 4). There was also no evidence for an improvement in model fit following inclusion of either region (Χ^2^ = 4.9, p = 0.29) or sample type (Χ^2^ = 0.9, *p* = 0.6) as fixed effects.

In total, *Salmonella* was isolated or detected in 145 goat samples and 47 (32.4%) of these were serotyped or serogrouped. Among the 47 isolates serotyped from goat samples, 19 (40.4%) were *Salmonella enterica* serovar Typhimurium, five (10.6%) were *Salmonella enterica* Typhi and two (4.3%) each of *Salmonella enterica* serovar Amersfoort, *Salmonella enterica* serovar Derby, *Salmonella enterica* serovar Poona, and untypeable *Salmonella* species (Table 7).

### 3.3 Typhoidal *Salmonella*

*Salmonella enterica* serovar Typhi isolates were reported from 201 samples in nine studies from Eastern, Northern, and Western African regions. Among the 201 *Salmonella enterica* serovar Typhi isolated or detected, 165 (82.1%) were from poultry gut samples from one study and 36 (17.9%) were from cattle, goat, pig, poultry, and sheep meat or organ samples.

*Salmonella enterica* serovar Paratyphi A were reported from 40 samples in five studies in Northern and Western African regions. Among the 40 *Salmonella enterica* serovar Paratyphi A isolated or detected, 33 (82.5%) were isolated from cattle, and poultry gut samples and 7 (17.5%) were isolated from cattle, poultry, and sheep meat or organ samples.

### 3.4 Risk of bias

Forty four of 246 studies (17.9%) had an overall low risk of bias, 178 (72.4%) had an overall moderate risk of bias, and 24 (9.8%) had an overall high risk of bias (S1 file - B). In regards to sample selection and transport, the number of days, months, or years over which a particular study took place was specified in 127 (51.6%) studies. The time from sampling to laboratory was specified as less than 4 h in 54 (22.0%) studies and unspecified in 181 (73.6%) studies. Temperature during transport was at refrigeration temperatures in 25 (10.2%) studies, chilled in 82 (33.3%) studies, and unspecified in 137 (55.7%) of studies. With regards to laboratory testing, the amount of any individual sample tested was specified in 182 (74.0%) of 246 studies. *Campylobacter* or *Salmonella* specific liquid and solid media were used in 150 (61.0%) of studies. *Campylobacter* were incubated microaerophilically or in a candle jar in 61 (83.6%) of the 73 studies focusing on that organism. *Campylobacter* and *Salmonella* were specified as incubated between 35 °C and 42 °C in 48 (65.8%) of 73 and 120 (64.5%) of 186 studies, respectively. The speciation methods of *Campylobacter* isolates were deemed to have low or moderate risk of bias in 50 (68.5%) of 73 studies. The typing methods for *Salmonella* isolates was deemed to have low or moderate risk of bias in 91 (48.9%) of 186 studies.

Forty-three (8.3%) of 518 articles were excluded at the full text screening stage for being in a language other than English.

## 4 Discussion

Our systematic review has demonstrated widespread prevalence of *Campylobacter* species and *Salmonella* serovars in food animal species or meat across Africa. Both *Campylobacter* and *Salmonella* were most prevalent among samples from poultry and pigs, and were less common among samples from ruminant livestock and other animals. *C. jejuni* was the most predominant *Campylobacter* species and *Salmonella enterica* serovar Typhimurium was the most commonly identified *Salmonella* serovar in African food animals and meat products.

*Campylobacter* and *Salmonella* were present in animal gut, external, and meat or organ animal samples, but patterns of carriage or contamination varied among host species. Pathogen contamination during slaughter and meat processing may be more important in some animal species than others. The finding that *Campylobacter* were more likely to be isolated or detected from poultry external samples than poultry gut samples may result from contamination of feathers and skin by gut contents of many different animals, both while alive and during slaughter. On-farm environmental sources, both inside and outside housing, waterways and wildlife, including wild birds, have been identified as sources of *Campylobacter* (Agunos et al., 2014). In contrast, *Salmonella* were significantly more likely to be isolated or detected in poultry gut samples than poultry external and meat or organ samples. This may be, in part, due to relative concentration of carriage of the two pathogens in poultry, which a report has shown to be >5 log cfu/g greater for *Campylobacter* than *Salmonella* in poultry faecal samples (FSANZ and the South Australian Research and Development Institute, 2010). Environmental contamination of cattle hides from multiple sources may explain why *Salmonella* were significantly more likely to be isolated or detected from cattle external samples than cattle gut samples. No cattle external samples were tested for *Campylobacter* to compare these findings. *Campylobacter* were significantly more likely to be isolated or detected from cattle gut samples than cattle meat or organ samples, while *Salmonella* were significantly more likely to be isolated or detected from goat meat or organ samples than goat gut samples. *Campylobacter* are micro-aerophilic organisms and sensitive to extra-intestinal environments (Cools et al., 2005). From extra-intestinal samples types, *Campylobacter* cells may be present but damaged and therefore not culturable in the laboratory. The difference for *Salmonella* isolation and detection between goats and cattle may be a result of differences in the slaughter and production, differences in sample consistency (particularly faecal samples) or handling of meat products. Goats are more easily hung for dressing than cattle, likely decreasing floor-to-carcass contact and therefore faecal contamination. Larger slaughter facilities in Africa have been shown to have greater incidence of *Salmonella,* at all stages of the slaughter process, than smaller facilities (Hassanien et al., 2006).

We have shown that *Campylobacter* were significantly more likely to be isolated or detected in Central African food animal samples than those from other African regions following adjustment for types of samples tested. The prevalence of *Campylobacter* detection was particularly high among Central African poultry and was significantly higher than that of other regions (Fig. 3). *Salmonella* were significantly more likely to be isolated or detected from Central and Western African food animal samples than the referent region of Northern Africa. Unlike for *Campylobacter*, no single food animal species contributed to the higher *Salmonella* prevalence in Central and Western Africa. Rather, it seems that the *Salmonella* prevalence across all food animal species drove the regional differences. The finding that Southern African studies isolated or detected significantly more *Campylobacter* in goat and sheep samples but significantly less *Campylobacter* in pig samples, than the referent region of Northern Africa, reinforces that the contamination or carriage may vary between animal species within the same region.

*Campylobacter jejuni* was the predominant *Campylobacter* species isolated from food animals in Africa. However, goat and sheep, and poultry samples from Central Africa and pig faecal samples and pork regardless of region of origin, had a higher prevalence of *C. coli*. The observation that *C. coli* predominates in pig and pork samples agrees with studies from North and South America (Cummings et al., 2018; Modolo et al., 1999; Varela et al., 2007), and Europe (Boes et al., 2005; Kempf et al., 2017) with varying predominance in Asia (Haruna et al., 2013; Carrique-Mas et al., 2014). Thermophilic *Campylobacter* species, *C. jejuni*, *C. coli*, *C. lari,* and *C. upsaliensis* accounted for the majority of campylobacters isolated. Thermophilic campylobacters cause the majority of human *Campylobacter* infection but non-thermophilic species also cause human illness (Lastovica and le Roux, 2000). *C. sputorum*, *C. hyointestinalis*, *C. faecalis*, and *C. fetus* are non-thermophilic *Campylobacter* species, and were identified in low numbers from African food animals. For non-thermophilic species underestimation is particularly likely where studies use an elevated temperature for isolation.

*Campylobacter* were not recovered from African camel samples. *Campylobacter* have been recovered from camel faecal and meat samples elsewhere (Mohammed et al., 2015; Rahimi et al., 2010). As so few camel samples were tested for *Campylobacter* (*n* = 3), there is a need for more sampling of camels in Africa.

*Salmonella enterica* serovar Typhimurium was the most commonly identified serovar in African food animals in our review. This contrasts with findings in a report from the WHO Global Salm-Surv database (Galanis et al., 2006); a now discontinued web-based country databank of *Salmonella* serovars from human and non-human sources. In the WHO report, *Salmonella enterica* serovar Typhimurium was not in the top five *Salmonella* serovars submitted from non-human sources from African countries between 2000 and 2002. Instead, *Salmonella enterica* serovar Anatum (16%), *Salmonella enterica* serovar Enteritidis (16%), *Salmonella enterica* serovar Corvalis (8%), *Salmonella enterica* serovar Amsterdam (8%), and *Salmonella enterica* serovar Braenderup (8%) were the most common from non-human sources across the continent (Galanis et al., 2006). This may result in bias of reporting of non-human *Salmonella* strains isolates to the databank, particularly if submissions came from sick animals by way of veterinary laboratories. It should be noted that the Global Salm-Surv database had only three African countries contributing non-human data, compared to 27 countries represented in our analyses. The Global Salm-Surv database was also more restricted in time, reporting contributed data between 2000 and 2002, compared to between 1953 and 2016 for our analyses.

Common *Salmonella enterica* serovars from pigs varied from those isolated from other food animals. *Salmonella enterica* serovar Hadar, *Salmonella enterica* serovar Saintpaul, and *Salmonella enterica* serovar Eastbourne were the three most frequently identified serovars. The low proportion of porcine *Salmonella enterica* serovar Typhimurium isolates contrasts with reports from Europe (Animal and Plant Health Agency (APHA), 2014, Hald et al., 2003, Pires et al., 2011), North America (Letellier et al., 1999), South America (Kich et al., 2011) and Asia (Amal et al., 2014; Thai et al., 2012) where *Salmonella enterica* serovar Typhimurium is a common serovar in pig and pig products.

Typhoidal and Paratyphoidal *Salmonella enterica* serovars Typhi, Paratyphi A, Paratyphi B, with the exception of the biovar Java, and Paratyphi C are not known to have animal or environmental reservoirs (Centers for Disease Control and Prevention (CDC), 2017). One *Salmonella* isolate reported as *Salmonella enterica* serovar Paratyphi B was from a study published in 1953, prior to *Salmonella enterica* serovar Java first being redefined as a biovar of *Salmonella enterica* serovar Paratyphi B in 1988 by Le Minor (Le Minor, 1988). The majority of the *Salmonella enterica* serovar Typhi reported as isolated or detected was from a single Western African poultry study whose typing methods were questionable and considered high risk for bias. However, three other studies from Western Africa reported *Salmonella enterica* serovar Typhi in poultry. Of 11 studies reporting the isolation of *Salmonella enterica* serovar Typhi from meat pathway samples, one (9.1%) reported farm production type, slaughter facility size, or characteristics of meat retail facilities. The isolation of *Salmonella enterica* serovar Typhi and *Salmonella enterica* serovar Paratyphi from gut, external and meat or organ sample types likely suggests contamination from human faeces indicating serious breaches in sample collection and processing, or extremely poor farm or slaughter facility hygiene practices.

We have shown that African food animals and meat may be an important source of campylobacteriosis and salmonellosis as has been shown worldwide (Barrow et al., 1988; Ellis, 1969; Sahin et al., 2002; Skarp et al., 2016). Source attribution studies have implicated chicken as a cause of >70% of human campylobacteriosis cases in studies from Germany (Rosner et al., 2017) and Switzerland (Kittl et al., 2013). Other studies report chicken consumption to be the predominant source of campylobacteriosis in other parts of Europe, Canada and New Zealand (Bessell et al., 2012; Boysen et al., 2011; Levesque et al., 2013; Mughini Gras et al., 2012; Ravel et al., 2017; Smid et al., 2013; Mullner et al., 2009). Ruminants appear to be the source of ~15–30% human campylobacteriosis in some studies, but as low as 1% in Germany (Rosner et al., 2017). Poultry was the most common source of foodborne salmonellosis in both the US and Japan where ~70% of human cases were attributable to chicken, turkey and egg products (Guo et al., 2011; Pires et al., 2014; Toyofuku and Hald, 2011). Poultry was similarly found to be the most important source of human salmonellosis in Europe. According to a European Food Safety Authority (EFSA) report, poultry was the greatest food animal contributor to human illness, with 51.2% of cases attributed to laying hens, broilers, and turkeys. We have shown that poultry in Africa have a higher prevalence of both *Campylobacter* and *Salmonella* than other food animals and meat suggesting that poultry may be an important source of human illness in Africa too. The second most common attributable source of illness in both the EU and Japan were pigs with 26.9% and 5.3% of cases respectively (Pires et al., 2011; Pires et al., 2014). Beef was the second most common attributable source in the US with 29% of cases (Guo et al., 2011).

While source attribution studies have been performed in some parts of the world, few are available for *Campylobacter* and *Salmonella* in Africa (Mather et al., 2015). It is important to ascertain whether or not increasing scales and intensification of meat production are contributing to human disease in Africa. The presence of *Campylobacter* and *Salmonella* in food animals and meat products may or may not indicate human disease risk. As we have shown, *Campylobacter* species and *Salmonella* serovars identified, vary between animal species. Without a more in depth look at the link between animal and human isolates, such as whole genome sequencing and patterns of human exposure, including raw meat handling and cooking practices, we have less information about whether or not *Campylobacter* and *Salmonella* from African food animals are contributing to human disease.

Foodborne diseases are ‘widespread and represent significant threats to health and economies of countries’ according to the WHO Regional Office for Africa (World Health Organisation (WHO) Regional Office for Africa, 2017). It has been shown that implementation of greater hygiene measures in slaughter systems has been successful in reducing the numbers of cases of salmonellosis in Europe (European Food Safety Authority (EFSA): European Centre for Disease Prevention Control, 2012) and campylobacteriosis in New Zealand (Cressey and Lake, 2011). There are many challenges to improving food safety in Africa. Some are logistical, such as reliable access to electricity and safe water, others are social, such as population changes, and some are environmental, such as extreme weather conditions (Food and Agriculture Organization of the United Nations (FAO) and World Health Organization (WHO), 2005). Where prevalence data show that end product samples are highly contaminated with *Campylobacter* or *Salmonella*, as was the case for poultry meat and organs, local and national policy makers and enforcers may be able to more effectively develop control measures to reduce potential pathogens in the food chain.

### 4.1 Limitations of the study

Our formal bias assessment determined the overall risk of bias from sample selection, sample transport and laboratory methods used. One hundred and eighty-two (73.7%) of 247 studies did not specify sample transport time, and 138 (55.9%) did not specify temperature of sample transport, both of which can affect the survival of pathogen before testing.

Most of the included studies either began testing samples in the years between 2000 and 2016 or did not specify when the study began. The analyses are therefore weighted more heavily on more recent studies as opposed to results uniformly spread over the years since 1951 when earliest sampling was stated to have taken place.

Laboratory methods varied between studies limiting direct comparability between analyses. Sixty-four (26.0%) of 247 studies did not specify the amount of sample tested. The weight of sample tested has been shown to influence the estimates of prevalence of *Salmonella* (Funk et al., 2000). Forty (16.2%) of 247 studies did not specify culture methods used. Among the 73 *Campylobacter* studies that used culture techniques for isolation, nine different broths were used as selective enrichment and 14 different agars were used. Among the 177 *Salmonella* studies that used culture techniques, eight different primary enrichment broths, six different selective enrichment broths, and 24 different agars were used for isolation. Speciating emerging *Campylobacter* requires tests beyond basic phenotypic and biochemical assays. The prevalence of these *Campylobacter* species is therefore likely underestimated. Prevalence estimates were not corrected for test sensitivity and specificity, implying that the true prevalence in each study is likely to differ from the apparent prevalence, and that the magnitude of this difference may be affected by sampling and testing methodology in an unquantified manner. No study was excluded due to chosen sampling or testing methodologies. Potential biases involving study size and sample type were adjusted for in the data analysis.

### 4.2 Conclusion

For many subsistence households and communities in many African countries, meat is a key protein source and livestock and poultry production is central to people's livelihoods. As market-driven changes occur within agricultural production towards wider distribution networks, centralised processing, and more large-scale and intensive systems, have been linked to the emergence of zoonotic diseases. Both *Campylobacter* and *Salmonella* were most prevalent among samples from poultry and pigs, and were less common among samples from ruminant livestock species. *C. jejuni* was the predominant *Campylobacter* species and *Salmonella enterica* serovar Typhimurium was the most commonly identified *Salmonella* serovar in African food animals and animal products. The presence of *Salmonella enterica* serovar Typhi and *Salmonella enterica* serovar Paratyphi in samples of food animal origin is particularly troubling and strongly indicates the need for increased hygiene measures to ensure food animals are not exposed to human faeces and human faeces do not contaminate the community meat supply.

The high prevalence of these organisms in livestock and poultry, their important role as human pathogens, and lack of evidence on which animal hosts contribute most to human illness in Africa, indicate source attribution studies would be a useful tool to more definitively identify priorities for food safety interventions.

The following are the supplementary data related to this article.

Supplementary File 1 PRISMA checklist. Supplementary File 1

Supplementary File 2 Inclusion and exclusion criteria. Supplementary File 2

Supplementary Table 1 Bias assessment of all included studies. Supplementary Table 1

Supplementary Table 2 Names and number of isolates for all *Salmonella enterica* serovars named in included articles according to African region. Supplementary Table 2

## Author contributions

All authors have contributed the concept or design of the work. KMT organised and populated the database. KMT wrote the first draft and subsequent revisions of the manuscript. WdG performed the meta-analysis. JAC, RNZ, NPF, JJB, SC, MAD, GB, ESS, JB, and WdG made substantial contributions to the manuscript. All authors contributed to manuscript revisions, read and approved the submitted version.

## Funding

The authors were funded by the UK Biotechnology and Biological Sciences Research Council, Department for International Development, UK, UK Economic and Social Research Council, UK Medical Research Council, UK Natural Environment Research Council and UK Defence Science and Technology Laboratory, under the Zoonoses and Emerging Livestock Systems (ZELS) programme (grant numbers BB/L017679/1 and BB/L018926/1). The funders were not involved in study design, analysis or interpretation of data, writing or decision to submit for publication.

## Declaration of competing interest

The authors declare that the submitted work was conducted in the absence of any commercial or financial relationships that could be construed as a conflict of interest.
