# Supplementary material for: Prevalence of Campylobacter and Salmonella in African food animals and meat: A systematic review and meta-analysis
Source: Int J Food Microbiol. 2020 Feb 16;315:108382. doi: 10.1016/j.ijfoodmicro.2019.108382 (PMC6985902; doi:10.1016/j.ijfoodmicro.2019.108382)
Supplement: Supplementary File 2 — Inclusion and exclusion criteria. [file mmc2.docx]

**A – *Inclusion and Exclusion Criteria***

**Study protocol to answer the question:**

What is the prevalence of *Campylobacter* and *Salmonella* in African food animals and animal food products?

**Relevance Tool - Screening Abstracts**

1. Is this primary research in English (or languages we can translate)? Y

N

1. Does this research investigate *Campylobacter spp.* and/or *Salmonella spp.*

in target animals/products? Y N

1. Does this study report on data from African countries or regions? Y

N

1. Is this an original study/article? Y

N

If the answers to questions 1, 2, 3 & 4 are Yes, then the article will be included for further screening and appraisal.

**Screening article content – full text review**

The major inclusion/exclusion criteria for full text review are:

1. Is the full text available? Y=include
2. (To confirm) Is the article about *Campylobacter* or *Salmonella*? Y=include
3. Is the article reporting on isolating/detecting these pathogens in individual food animals/products? Y=include

- In regards to faecal samples, it should be clear that the sample is from different animals to prevent sampling from the same animal multiple times (e.g. freshly voided are OK if samples are clearly from different animals).

1. Is the prevalence of the pathogen reported as a percentage of the animal sample (or can you figure it out from the information given)? Y=include
2. Are the samples tested relevant (faeces, cloaca, caeca, intestinal contents, hide, carcass, raw meat, raw offal, edible unprocessed by-products, lymph nodes)? Y=include

- Unadulterated minced meat can be included. Samples such as sausages are excluded on the basis that additives to the meat could be the source of contamination.

1. (To confirm) Does this study report on data from Africa countries or regions? Y=include

- Country/location where samples were obtained or where samples were processed should be stated somewhere in the abstract or the body of the text.

Criteria which came up during the full text review:

1. Are the samples frozen at origin? Y=exclude

- Considered as part of the method if samples are frozen in the lab before testing, therefore incorrect sample type.

1. Are samples pooled? Y=exclude

- If pooled samples are returned to individually to find individual prevalence or there is a calculation made to estimate individual prevalence then study can be included. Slurry samples are considered to be pooled or environmental samples.

1. Is the study testing samples solely from sick animals? Y=exclude
2. Is the study solely investigating wildlife or game? Y=exclude

- Animals should obviously belong to someone rather than, for example, sampling faeces of non-domestic ducks around an open community lake which can be considered to be more like environmental sampling.

1. Is the study solely investigating environmental or human samples? Y=exclude

If the answers to all questions 5 – 15 indicate inclusion then the article/study data will be included in systematic review.
