# Supplementary material for: Prevalence of Campylobacter and Salmonella in African food animals and meat: A systematic review and meta-analysis
Source: Int J Food Microbiol. 2020 Feb 16;315:108382. doi: 10.1016/j.ijfoodmicro.2019.108382 (PMC6985902; doi:10.1016/j.ijfoodmicro.2019.108382)
Supplement: Supplementary Table 1 — Bias assessment of all included studies. [file mmc3.docx]

**Supplementary Table 1 - *Quality assessment***

| **Author, Year** | Study time period introducing bias | Reason for the study biasing results | Impact of transport to lab | | Amount of sample tested known | Bias generated by quality of detection methods used | *Campylobacter* Bias from incubation conditions | *Salmonella* Bias from incubation conditions | *Campylobacter* Quality of typing methods bias | *Salmonella* Quality of typing methods bias | **Overall assessment of risk of bias** |
| --- | --- | --- | --- | --- | --- | --- | --- | --- | --- | --- | --- |
|  |  |  | Time | Temperature |  |  |  |  |  |  |  |
|  | Selection and sample handling | | | | Laboratory testing | | | | | |  |
| A Mpalang, R. K. B.,2014 | L | L | L | L | Y | L | L | NA | L | NA | L |
| Abrahams, C. A. A.,1990 | U | L | U | U | Y | M | L | NA | H | NA | M |
| Adekeye, J. O. A.,1989 | U | L | L | U | Y | L | L | NA | L | NA | L |
| Akwuobu, C. A. O.,2010 | U | L | L | U | Y | M | L | NA | M | NA | M |
| Awadallah, M. A. I. A.,2014 | L | L | U | U | Y | L | L | NA | L | NA | L |
| Bester, L. A.,2012 | U | L | U | U | N | M | I | NA | M | NA | M |
| Cardinale, E. D.,2003 | L | L | U | U | N | U | I | NA | U | NA | M |
| Cardinale, E. R.,2006 | L | L | U | U | N | U | I | NA | L/M | NA | M |
| Chanyalew, Y. A.,2013 | M | L | U | M | Y | M | L | NA | M | NA | M |
| Cooper, R. S.,2001 | U | M | U | U | Y | U | I | NA | H | NA | M |
| Dadi, L. A.,2008 | M | L | U | M | Y | L | L | NA | M | NA | M |
| El-Gamal Galal, B.,1992 | U | L | U | U | Y | L | L | NA | U | NA | M |
| El-Jakee, J.,2015 | U | L | L | U | Y | L | L | NA | L | NA | L |
| El-Shibiny, A. A. S.,2012 | U | M | U | U | N | M | L | NA | NA | NA | M |
| El-Tras, W. F. H.,2015 | L | M | U | M | Y | M | L | NA | M | NA | M |
| Elegbe, I. A.,1987 | U | L | U | U | N | M | L | NA | M | NA | M |
| Ewnetu, D.,2010 | M | L | U | M | Y | M | L | NA | M | NA | M |
| Garin, B.,2012 | L | L | L | L | Y | L | L | NA | M | NA | L |
| Gblossi Bernadette, G. E. E.,2012 | M | L | U | M | Y | M | L | NA | L | NA | M |
| Goualie, G. K.,2007 | H | L | U | U | N | M | L | NA | U | NA | M |
| Hassanain, N. A.,2011 | L | L | U | M | N | M | L | NA | M | NA | M |
| Jacob, P.,2011 | U | M | L | M | Y | M | M | NA | M | NA | M |
| Jiwa, S. F. H. K.,1994 | U | L | L | L | N | M | L | NA | U | NA | M |
| Jonker, A. P.,2010 | M | L | L | M | N | M | L | NA | M | NA | M |
| Kashoma, I. P.,2015 (6?) | L | L | H | L | Y | L | L | NA | L | NA | H |
| Kashoma, I. P.,2015 | L | L | H | M | Y | L | L | NA | L | NA | M |
| Kassa, T.,2005 | M | L | U | U | Y | M | L | NA | M | NA | M |
| Khadr, A. M. H.,2006 | U | L | U | U | Y | M | L | NA | M | NA | M |
| Khalafalla, F. A.,1990 | U | L | U | U | Y | L | M | NA | M/H | NA | M |
| Khalifa, N. O.,2013 | U | L | U | M | Y | L | L | NA | L | NA | M |
| Komba, E. V. G. M.,2014 | U | L | U | M | Y | L | L | NA | L | NA | M |
| Mabote, K. I. M.,2011 | U | L | U | U | Y | M | H | NA | M | NA | H |
| Mdegela, R. H. N.,2006 | L | L | U | U | Y | L | M | NA | U | NA | M |
| Mdegela, R. H. N.,2007 | U | L | U | U | Y | U | I | NA | L/M | NA | M |
| Mdegela, R. H.,2011 | U | L | L | L | Y | L | M | NA | M | NA | M |
| Messad, S.,2014 | L | L | U | L | Y | M | L | NA | M | NA | M |
| Montwedi, M. A.,2012 | U | L | U | U | Y | M | M | NA | H | NA | H |
| Ngulukun, S. S. O., 2010 | H | L | U | M | U | L | L | NA | L | NA | M |
| Ngulukun, S. S. O.,2011 | L | L | U | M | Y | L | L | NA | L | NA | L |
| Nigatu, S.,2015 | M | L | U | U | Y | M | L | NA | H | NA | M |
| Nonga, H. E. M.,2010 | M | L | U | U | Y | L | M | NA | M | NA | M |
| Nonga, H. E. S.,2009 | U | L | L | L | Y | L | M | NA | M | NA | L |
| Ofukwu, R. A.,2008 | M | L | U | U | Y | M | L | NA | M/H | NA | M |
| Okunlade, A. O. O.,2015 | U | L | M | M | Y | M | L | NA | M | NA | M |
| Olubunmi, P. A.,1986 | U | L | L | U | Y | M | L | NA | U | NA | M |
| Omara, S. T. E. F.,2015 | L | L | U | U | N | L | L | NA | M/H | NA | M |
| Osano, O. A.,1999 | H | L | L | M | Y | L | L | NA | L | NA | L |
| Raji, M. A.,2000 | L | L | L | U | N | M | M | NA | M | NA | M |
| Ramadan, H. J.,2015 | M | L | L | U | N | H | NA | NA | L | NA | H |
| Richardson, N. J. K.,1979 | U | M | L | U | Y | H | M | NA | H/U | NA | H |
| Salihu, M. D. A.,2009 | L | L | M | U | Y | M | L | NA | M | NA | M |
| Salihu, M. D. A.,2009 | L | L | L | U | Y | M | L | NA | M | NA | L |
| Salihu, M. D.,2012 | H | L | L | M | Y | M | L | NA | M | NA | M |
| Turkson, P. K. L.,1988 | U | L | L | U | Y | M | M | NA | M | NA | M |
| Uaboi-Egbenni, P. O. B,2011 | U | L | U | L | Y | M | L | NA | M | NA | M |
| Uaboi-Egbenni, P. O. B.,2010 | U | L | L | U | Y | M | L | NA | M | NA | M |
| Uaboi-Egbenni, P. O. B.,2011 | U | L | L | M | Y | M | L | NA | M | NA | M |
| Uaboi-Egbenni, P. O. B.,2012 | U | L | L | M | Y | M | L | NA | M | NA | M |
| Uaboi-Egbenni, P. O. O,2008 | U | L | U | U | N | M | L | NA | M | NA | M |
| Woldemariam, T. A.,2009 | M | L | U | M | Y | M | L | NA | M | NA | M |
| Abd el-Aziz, A. S. E.,2002 | U | L | U | U | N | L | M | L | M | M | M |
| Acha, S. J. K.,2004 | L | L | U | U | N | L | L | L | M | M | M |
| Ali, F. H. M.,2005 | U | M | U | M | Y | L | I | L/M | U | U | M |
| Cardinale, E. G.-C.,2003 | L | L | U | L | Y | L | L | L | M | M | L |
| Elegbe, I. A.,1983 | M | L | L | U | Y | M | L | L | M | U | L |
| Khalafalla, F. A.,2015 | U | L | U | U | N | U | I | I | U | U | M |
| Kusiluka, L. J. M. K.,2005 | M | L | U | M | N | L | M | I | M | NA | M |
| Nassar, A. M.,2000 | U | L | L | U | N | L | L | L | H | U | M |
| Nzouankeu, A. N.,2010 | L | L | L | M | Y | U | I | I | U | M | M |
| Rady, E. M.,2011 | U | L | U | U | Y | U | I | I | U | NA | M |
| Sackey, B. A. M.,2001 | U | L/M | U | M | Y | L | M | M | M | M | M |
| Samaha, I. A.,2012 | U | L | U | U | U | L | I | I | H | NA | H |
| van Nierop, W.,2005 | U | L | L | M | N | L | L | L | M | M | M |
| Abbassi-Ghozzi, I. J.,2012 | L | L | U | M | Y | L | NA | L | NA | L | L |
| Abd El-Ghany, W. A. E.-S.,2012 | U | M | U | M | Y | L | NA | L | NA | L | M |
| Abd-Elghany, S. M.,2015 | M | L | L | L | N | M | NA | I | NA | M | M |
| Abdel-Maksoud, M.,2015 | M | M | L | U | I | U | NA | U | NA | H | H |
| Abdellah, C. F.,2008 | L | L | U | M | Y | L | NA | L | NA | M | L |
| Abebe, M.,2014 | M | L | U | M | Y | L | NA | L | NA | U | M |
| Abou El Hassan, D. G.,1996 | U | M | L | M | Y | L | NA | U | NA | NA | M |
| Addis, Z. K.,2011 | M | L | U | M | Y | L | NA | L | NA | NA | M |
| Adesiji, Y. O. A.,2011 | M | L | L | M | Y | H | NA | L | NA | NA | H |
| Adesiyun, A. A. O.,1989 | U | L | U | U | Y | L | NA | L | NA | L | M |
| Adeyanju, G. T.,2014 | M | L | U | U | Y | L | NA | L | NA | NA | M |
| Adu-Gyamfi, A.,2012 | U | L | U | M | Y | M | NA | H | NA | NA | H |
| Ahmed, A. M.,2014 | L | L | U | M | Y | L | NA | U | NA | H | H |
| Ahmed, H. A.,2014 | M | L | U | U | Y | U | NA | U | NA | H | H |
| Ahmed, H. A.,2016 | U | L | U | U | I | U | NA | U | NA | M/H | M |
| Ajayi, A. O. E., 2011 | U | L | U | U | Y | M | NA | L | NA | H | H |
| Akam, A. K.,2004 | U | M | U | L | N | U | NA | U | NA | NA | M |
| Akoachere, J.-F. T. K. T.,2009 | M | L | U | U | Y | L | NA | U | NA | H | H |
| Al-Hazmi, M. A.-A.,2013 | U | L | U | M | Y | U | NA | U | NA | M/H | M |
| Alao, F. O. K.,2012 | U | L | L | M | Y | L | NA | L | NA | H | H |
| Alemayehu, D. M.,2003 | M | L | U | U | Y | L | NA | L | NA | L | M |
| Alemu, S. Z.,2012 | M | L | U | L | Y | L | NA | L | NA | L | L |
| Aliaa, S. O.,2014 | U | L/M | U | M | Y | U | NA | U | NA | NA | M |
| Amal, A. A. S.,2014 | U | L | U | U | Y | L | NA | M | NA | M | M |
| Amara, A.,1994 | U | L | U | U | Y | U | NA | U | NA | NA | M |
| Amin, H. S.,2015 | U | M | U | M | I | U | NA | U | NA | L | M |
| Ammar, A. A.,2010 | U | M | L | U | I | L | NA | L | NA | M | M |
| Ammar, A. M. A.,2010 | U | M | U | U | Y | U | NA | U | NA | H | H |
| Anbessa, D.,2012 | M | L | U | M | Y | L | NA | L | NA | NA | M |
| Aragaw, K. M.,2007 | M | L | U | M | Y | U | NA | I | NA | L | M |
| Aragaw, K.,2010 | M | M | U | M | Y | M | NA | M | NA | H | H |
| Ashenafi, M.,1994 | U | L/M | L | U | Y | L | NA | L | NA | NA | M |
| Bada-Alambedji, R. F.,2006 | M | L/M | U | M | Y | L | NA | L | NA | L | M |
| Bata, S. I.,2016 | U | L | U | U | Y | M | NA | L | NA | NA | M |
| Bawa, I. H.,2015 | L | L | L | L | Y | L | NA | L | NA | NA | L |
| Bekele, B. A.,2010 | U | L | U | M | Y/N | L | NA | U | NA | NA | M |
| Beshatu, F.,2015 | M | L | U | M | Y | L | NA | L | NA | NA | M |
| Boko, C. K. K.,2013 | U | M | U | U | Y | L | NA | L | NA | M | M |
| Bouchrif, B. P.,2009 | H | L | U | L | Y | L | NA | L | NA | M | L |
| Chambers, P. G.,1977 | M | L | U | U | Y/N | L | NA | L | NA | L | M |
| Cohen, N. E.,2007 | L | L | L | L | Y | U | NA | I | NA | NA | L |
| Cohen, N. E.,2006 | L | L | L | L | Y | L | NA | L | NA | NA | L |
| Collard, P.,1956 | M | U | U | U | Y/N | L | NA | U | NA | L | M |
| Dabassa, A.,2013 | M | L | L | U | Y | L | NA | L | NA | NA | L |
| Daniyan, S. Y.,2011 | H | L | U | H | Y | L | NA | L | NA | NA | H |
| Dione, M. M.,2009 | U | L | U | M | I | L | NA | L | NA | L | M |
| Dione, M. M.,2011 | L | M/H | M | U | Y | M | NA | L | NA | M | M |
| Eguale, T.,2016 | M | L | L | M | Y | L | NA | L | NA | L | L |
| Eisa, M. I.,2004 | U | M | U | U | Y | L/M | NA | L | NA | M | M |
| Eissa, W. M. M.,2014 | U | L | U | U | Y | L | NA | L | NA | NA | M |
| Ejeta, G. M.,2004 | M | L | U | U | Y | L | NA | L | NA | L | M |
| El Naker, Y. F. I.,2007 | L | H | U | U | Y | L | NA | L | NA | U | H |
| El-Gamal, A. M.,2016 | U | L | U | U | Y | L | NA | L | NA | M | M |
| El-Tras, W. F. T.,2010 | L | M | U | U | Y | M | NA | L | NA | H | H |
| Elmossalami, E. Y.,1994 | U | L | U | U | I | L | NA | M | NA | M | M |
| Falade, S. E.,1981 | U | L | U | U | Y | L | NA | L/U | NA | L | M |
| Farrag, H. E.-A.,1954 | U | L | U | U | N | L | NA | L | NA | U | M |
| Farrag, H. F. E.-G.,1962 | U | L | U | U | N | L | NA | L/U | NA | U | M |
| Farrag, H.,1956 | U | L | U | U | I | L | NA | L | NA | U | M |
| Fashae, K. O.,2010 | U | L | U | U | Y | L | NA | L | NA | L | M |
| Floyd, T. M. B.,1953 | H | L | L | U | Y | L | NA | L/U | NA | L | M |
| Gaedirelwe, O. G. S.,2008 | U | L | U | M | Y | L | NA | L | NA | NA | M |
| Garedew, L. H.,2015 | M | L | U | M | Y | M | NA | L | NA | NA | M |
| Gashe, B. A.,2000 | L | L | L | U | Y | L | NA | L | NA | NA | L |
| Gebeyehu, A.,2013 | U | M | U | U | Y | L | NA | L | NA | NA | M |
| Gharieb, R. M.,2015 | L | L | U | M | Y | L | NA | L | NA | M | L |
| Ghoneim, N. H. M.,2015 | U | L | U | U | N | L | NA | L/U | NA | M | M |
| Gitter, M. B.,1970 | U | L | U | M | I | L | NA | L/M | NA | U | M |
| Gopo, J. M.,1997 | U | L | U | U | Y/N | U | NA | L | NA | NA | M |
| Guergueb, N.,2014 | M | L | U | U | I | U | NA | I | NA | NA | M |
| Hag Elsafi, H. E. N. E.,2009 | U | L | U | U | I | U | NA | I | NA | L | M |
| Hamada, S.,1963 | L | L | U | U | Y | L | NA | L | NA | M | L |
| Hang'ombe, B. M. S.,1999 | H | L | U | U | Y | L | NA | L | NA | M | M |
| Hassb-Elnaby, G. R.,2011 | M | M | U | U | N | U | NA | U | NA | U | M |
| Hughes, F. A. A.-G.,2015 | U | M | U | U | Y | M | NA | L | NA | NA | M |
| Hummel, P. H.,1974 | M | L | U | U | Y | L | NA | L | NA | L/M | M |
| Ibrahim, A. E.,1974 | U | L | U | U | Y | L | NA | U | NA | L | M |
| Ibrahim, M. A.,2016 | L | L | L | M | Y | L | NA | L | NA | L | L |
| Ikwap, K.,2014 | U | L | U | M | Y | L | NA | L | NA | L | M |
| Iroha, I. R.,2011 | M | L | U | U | Y | U | NA | L/U | NA | H | M |
| Isogai, E. S.,2005 | U | L | U | U | Y | M | NA | L | NA | NA | M |
| Iwu, C. J.,2016 | U | L | U | U | I | L | NA | L | NA | NA | M |
| Jajere, S. M.,2015 | U | L | M | L | Y | L | NA | L | NA | NA | L |
| Kagambega, A. B.,2012 | M | L | L | M | Y | L | NA | L/U | NA | M | M |
| Kagambega, A. H.,2011 | M | L | L | M | Y | L | NA | L | NA | L | L |
| Kagambega, A. L.,2013 | M | L | M | U | Y | L | NA | L | NA | M | M |
| Kapondorah, T. L. S.,2007 | H | L | U | U | Y | L | NA | L | NA | NA | M |
| Khallaf, M. A.,2014 | L | L | M | L | Y | L | NA | L | NA | M | L |
| Khan, A. Q.1970 | U | L | U | U | N | L | NA | L | NA | L/M | M |
| Khan, A. Q.1970 | U | L | U | U | N | L | NA | L | NA | M | M |
| Khan, A. Q.1970 | U | L | U | U | N | L | NA | L | NA | M | M |
| Kikuvi, G. M.,2007 | M | L | U | M | Y | L | NA | L | NA | L | M |
| Kpodekon, M. T. G.,2013 | U | L | U | M | Y | L | NA | L | NA | NA | M |
| Kuroda, K. S.,2013 | U | L | U | U | N | NA | NA | L | NA | NA | M |
| Kwaga, J. K.,1985 | U | L | U | U | Y | M | NA | L | NA | NA | M |
| Lotfi, Z. S.,1964 (buffalo) | L | L | U | U | Y | M | NA | L | NA | M | M |
| Lotfi, Z. S.,1964 | L | L | U | U | Y | M | NA | L | NA | M | M |
| Madoroba, E.,2016 | L | L | U | M | Y | L | NA | L | NA | M/H | L |
| Mahangaiko, M.,2015 | U | L | U | M | Y | L | NA | U | NA | NA | M |
| Mahmoud, Y. E.,2006 | U | L | U | M | Y | L | NA | L | NA | M | M |
| Mathole, M. A.,2016 | L | L | U | U | Y | U | NA | I | NA | M | M |
| Mdegela, R. H. Y.,2000 | L | M | U | U | Y | L | NA | L | NA | H | H |
| Meara, P. J. M.,1977 | U | L | U | U | Y | U | NA | I | NA | L | M |
| Mezali, L. H.,2012 | L | L | U | M | Y | L | NA | L | NA | M | L |
| Miller, A. S.,1971 | L/U | L | U | U | N | L | NA | L | NA | U | M |
| Mira, E. K. I. E.,2007 | U | L | U | U | I | U | NA | I | NA | L | M |
| Missohou, A.,2011 | L | L | U | U | I | L | NA | L | NA | NA | M |
| Moawad, R. K.,2013 | U | M | U | M | I | M | NA | L | NA | NA | M |
| Mohamed, I. A. H., 2014 | M | L | L | M | Y | L | NA | L | NA | M | L |
| Mohamed, S. R.,2007 | U | L | U | U | N | L | NA | L | NA | H | H |
| Mohammed, M. E. H., 2003 | H | H | U | M | Y | L | NA | U | NA | NA | H |
| Molla, B. B.,2006 | M | L | U | U | Y | L | NA | L | NA | L | M |
| Molla, B. K.,1999 | U | L | U | U | I | U | NA | I | NA | U | M |
| Molla, B. M.,2003 | M | L | U | M | Y | L | NA | L | NA | M | M |
| Molla, B. M.,2004 | M | L | U | M | Y | L | NA | L | NA | L | M |
| Molla, B.,2003 | L | L | U | M | Y | L | NA | L | NA | L | L |
| Motsoela, C. C.,2002 | L | L | U | U | Y | L | NA | L | NA | M | L |
| Mousa, M. M. E. B.,2009 | U | M | U | U | I | U | NA | I | NA | U | M |
| Moussa, I. M. H.,2014 | U | L | U | L | Y | U | NA | I | NA | M | M |
| Moussa, I. M.,2013 | L | L | U | U | Y | L | NA | L | NA | M | L |
| Muluneh, G. K.,2015 | H | L | L | L | Y | L | NA | L | NA | NA | M |
| Nabawy, E. E.,2016 | M | L | U | M | I | U | NA | I | NA | M | M |
| Nawar, E. M.,2014 | U | L | U | U | Y | L | NA | L | NA | M | M |
| Ngoma, M. P.,1996 | U | L | U | U | Y | L | NA | L | NA | M | M |
| Niyonzima, E.,2016 | U | L | U | U | Y | L | NA | I | NA | NA | M |
| Nossair, M. A.,2015 | U | M | U | M | Y | L | NA | I | NA | M | M |
| Nouichi, S. H.,2009 | M | L | U | M | Y | L | NA | L | NA | U | M |
| Nwachukwu, N. C. O.,2010 | U | L | L | U | Y | L | NA | L | NA | NA | L |
| Nyamakwere, F.,2016 | M | L | U | M | Y | L | NA | L | NA | NA | M |
| Nyeleti, C. H.,2000 | U | L | U | U | Y | L | NA | I | NA | M/U | M |
| Oboegbulem, S. I. M.,1981 | U | L | U | U | N | L/U | NA | I | NA | L | M |
| Ola Ojo, M.,1974 | U | L | U | U | Y | L | NA | L | NA | L | M |
| Olatoye, O. I.,2011 | H | L | U | M | Y | L | NA | L | NA | NA | M |
| Olayemi, A. B. G.,1979 | M | L | U | U | N | L | NA | L | NA | L | M |
| Oluyege, A. O. O.-B.,2015 | M | L | L | U | Y | L | NA | I | NA | NA | M |
| Onyekaba, C. O.,1986 | L | L | U | U | Y | M | NA | M | NA | U | M |
| Orji, M. U. O.,2005 | M | L | L | U | Y | L | NA | L | NA | U | L |
| Osman, K. M. M.,2014 | U | L | L | L | Y | L | NA | L | NA | M | L |
| Osman, K. M. M.,2014 | U | L | M | M | Y | U | NA | I | NA | M | M |
| Osman, K. M. M.,2014 | U | L | L | L | Y | L | NA | L | NA | M | L |
| Osman, K. M. Y.,2010 | U | L | L | L | N | L | NA | L | NA | M | M |
| Otaru, M. M. M. N.,1990 | L | L | U | U | Y | L | NA | L | NA | L | L |
| Oueslati, W.,2016 | L | L | U | U | Y | L | NA | L | NA | L | L |
| Ouf, J. M.,2004 | U | L | U | M | N | L | NA | M | NA | NA | M |
| Phagoo, L.,2015 | U | L | H | U | Y | L | NA | L | NA | NA | H |
| Prior, B. A.1974 | L | L | U | U | Y | U | NA | M | NA | L | M |
| Randa, G. A. S.,2014 | U | L | U | U | N | U | NA | I | NA | NA | M |
| Raufu, I. H.,2009 | H | L | U | U | N | L | NA | L | NA | L | M |
| Raufu, I.,2013 | L | L | U | U | Y | L | NA | L | NA | L | L |
| Refai, M., 1984 | U | L | U | U | N | L | NA | L | NA | U | M |
| Rene, K. A.,2014 | H | L | L | L | N | U | NA | I | NA | M | M |
| Richardson, N. J. B.,1968 | L | L | U | U | Y | L | NA | U | NA | U | M |
| Sallam, K. I. M.,2014 | U | L | U | M | Y | L | NA | L | NA | U | M |
| Samaha, H. A.,2002 | U | L | U | U | Y | U | NA | I | NA | M | M |
| Samaha, I. A.,2011 | U | L | U | M | Y | U | NA | I | NA | U | M |
| Scharawe, H. I. I.,2009 | U | L | U | U | Y | L | NA | L | NA | M | M |
| Sen, R.,1957 (fowl) | U | L | U | U | Y | U | NA | U | NA | L | M |
| Sen, R.,1957 (pigs) | H | L | U | U | Y | U | NA | I | NA | L | M |
| Shaltout, F. A. A.-A.,2004 | U | L | U | M | Y | L | NA | U | NA | M/H | M |
| Shilangale, R. P. K.,2015 | L | L | U | L | Y | L | NA | L | NA | L | L |
| Sibhat, B. M. Z.,2011 | M | L | U | M | Y | L | NA | L | NA | M | M |
| Smith, S. I. B.,2009 | L | L/M | L | U | Y | L | NA | L | NA | U | M |
| Smith, S.,2016 | M | L | L | H | Y | U | NA | U | NA | L | H |
| Stevens, A.,2006 | M | L | M | U | Y | L | NA | L | NA | L | M |
| Tafida, S. Y. K.,2013 | L | L | U | M | Y | L | NA | L | NA | L | L |
| Tanih, N. F. S.,2015 | U | L | L | U | Y | L | NA | L | NA | NA | L |
| Teklu, A.,2011 | M | L | U | M | N | L | NA | L | NA | NA | M |
| Tibaijuka, B. M.,2002 | M | L | U | M | Y | L | NA | L | NA | L | M |
| Wesonga, S. M. M.,2010 | U | L | U | U | Y | M | NA | L | NA | U | M |
| Woldemariam, E. M.,2005 | M | L | U | M | Y | L | NA | L | NA | L | M |
| Yagoub, I. A. M.,1987 | L | L | U | U | Y | L | NA | L | NA | L | L |
| Yizengaw, H. A.,2015 | U | L | U | U | Y | U | NA | L | NA | NA | M |
| Zahran, R. E.-B.,2014 | U | L | U | M | Y | L | NA | L | NA | M | M |
| Zishiri, O. T.,2016 | M | L | U | M | N | L | NA | L | NA | NA | M |

Low (L), Moderate (M), High (H), Yes (Y), No (N), Unknown (U), Implied (I), Not applicable (NA)
